# Anti-PD-L1 peptide-conjugated prodrug nanoparticles for targeted cancer immunotherapy combining PD-L1 blockade with immunogenic cell death

**DOI:** 10.7150/thno.69119

**Published:** 2022-01-31

**Authors:** Yujeong Moon, Man Kyu Shim, Jiwoong Choi, Suah Yang, Jinseong Kim, Wan Su Yun, Hanhee Cho, Jung Yeon Park, Yongju Kim, Joon-Kyung Seong, Kwangmeyung Kim

**Affiliations:** 1 Biomedical Research Institute, Korea Institute of Science and Technology (KIST), Seoul, 02792, Republic of Korea.; 2 Department of Bioengineering, Korea University, Seoul, 02841, Republic of Korea.; 3 KU-KIST Graduate School of Converging Science and Technology, Korea University, Seoul, 02841, Republic of Korea.; 4 Department of Materials Science and Engineering, Seoul National University, Seoul, 08826, Republic of Korea.

**Keywords:** cancer immunotherapy, immune checkpoint blockade, prodrug nanoparticle, drug delivery, antitumor immune response

## Abstract

**Rationale:** Cancer immunotherapy combining immune checkpoint blockade (ICB) with chemotherapeutic drugs has provided significant clinical advances. However, such combination therapeutic regimen has suffered from severe toxicity of both drugs and low response rate of patients. In this study, we propose anti-PD-L1 peptide-conjugated prodrug nanoparticles (PD-NPs) to overcome these obstacles of current cancer immunotherapy.

**Methods:** The functional peptide, consisted of anti-PD-L1 peptide and cathepsin B-specific cleavable peptide, is conjugated to a doxorubicin (DOX), resulting in prodrug nanoparticles of PD-NPs *via* intermolecular interactions. The antitumor efficacy and immune responses with minimal side effects by PD-NPs combining PD-L1 blockade and ICD are evaluated in breast tumor models.

**Results**: The PD-NPs are taken up by PD-L1 receptor-mediated endocytosis and then induce ICD in cancer cells by DOX release. Concurrently, PD-L1 blockade by PD-NPs disrupt the immune-suppressing pathway of cancer cells, resulting in proliferation and reinvigoration of T lymphocytes. In tumor models, PD-NPs accumulate within tumor tissues *via* enhanced permeability and retention (EPR) effect and induce immune-responsive tumors by recruiting a large amount of immune cells.

**Conclusions:** Collectively, targeted tumor delivery of anti-PD-L1 peptide and DOX *via* PD-NPs efficiently inhibit tumor progression with minimal side effects.

## Introduction

Immune checkpoint blockade (ICB) immunotherapy has provided significant clinical advances to combat a variety of cancers, which provokes an antitumor immune response *via* monoclonal antibodies (mAbs) against programmed cell death protein 1 (PD-1), programmed death-ligand 1 (PD-L1) or cytotoxic T-lymphocyte-associated protein 4 (CTLA-4) 1. Furthermore, the therapeutic outcomes of ICB immunotherapy have dramatically improved when combined with immunogenic cell death (ICD)-inducing chemotherapeutic drugs in pre-clinical and clinical studies 2. The underlying mechanism is that dying cells by ICD-inducing chemotherapeutic drugs release damage-associated molecular patterns (DAMPs) to promote the cross-presentation of tumor-associated antigens to the dendritic cells (DCs), and thus provokes DC maturation and T lymphocyte infiltration 3-6. These cascade events of ICD turn the immunosuppressive tumor microenvironment (ITM) to the immune-responsive tumors, leading to increase the ICB immunotherapy efficiency 6, 7. However, poor cancer-specificity of chemotherapeutic drugs can induce systemic toxicities as well as bone marrow suppression and damage to the host immune system, resulting in serious side effects and low response rate of patients 8. In addition, immunogenicity of mAbs for ICB immunotherapy increase the risk of uncontrolled autoimmune toxicities, the so-called immune-related adverse events (irAEs), which are unpredictable, possibly permanent and occasionally fatal in almost every organ 9, 10.

Due to the potential ability to block the PD-1/PD-L1 interactions, small molecular PD-1/PD-L1 binding peptides have been received much attention in cancer immunotherapy 11. For example, small peptide, NP-12, was reported as an anti-PD-1 peptide with 29 amino acid sequence to synergize with ICD for effective ICB immunotherapy 12. In addition, antagonist peptide of PD-L1 (CLQKTPKQC) effectively blocked the PD-1/PD-L1 interactions *via* high binding affinity to PD-L1 and reinvigorated the T lymphocytes, thereby inducing potent ICB immunotherapy when combined with ICD-inducing chemotherapeutic drugs 13. However, such PD-1/PD-L1 binding peptides have shown unfavorable therapeutic efficacy owing to the extensive proteolytic cleavage and short half-lives *in vivo* 14. To overcome these drawbacks, anti-PD-1 or anti-PD-L1 peptides have broadly adopted to multifunctional nanomedicines, such as polymeric nanoparticles, liposomes and dendrimers 15-18. Compared to mAbs for blockade of PD-1/PD-L1 interactions, these peptide antagonists have some advantages of amenability to chemical synthesis for design of various nanomedicine loading accurate contents due to their modifiable structure, and low production cost 16. In particular, anti-PD-1/anti-PD-L1 peptide-modified nanoparticles containing chemotherapeutic drugs elicited potent antitumor immune responses by inducing ICD and disruption of PD-1/PD-L1 interactions 19. The PD-1/PD-L1 blockade can remove the inhibitory signals for T lymphocyte activation and increase the T cell's recognition of cancer cells damaged by drugs, thereby eradicating heterogeneous and drug-resistant cancer cells by long-term durable and systemic immune responses 20. However, their translation into the clinic is still difficult because of the innate features of nanoparticle or delivery carrier itself. Many preclinical studies have shown that tumor-targeting nanomedicines have unexpectedly poor delivery efficiency (> 1-3%) and non-specific drug release into the normal tissues and blood stream raise the risk of organ dysfunction and systemic immunosuppression 21. Also, low drug loading efficiency, innate toxicity and difficulty in mass production of nano-sized carriers hinder their commercialization for ICB immunotherapy.

**Scheme 1:**
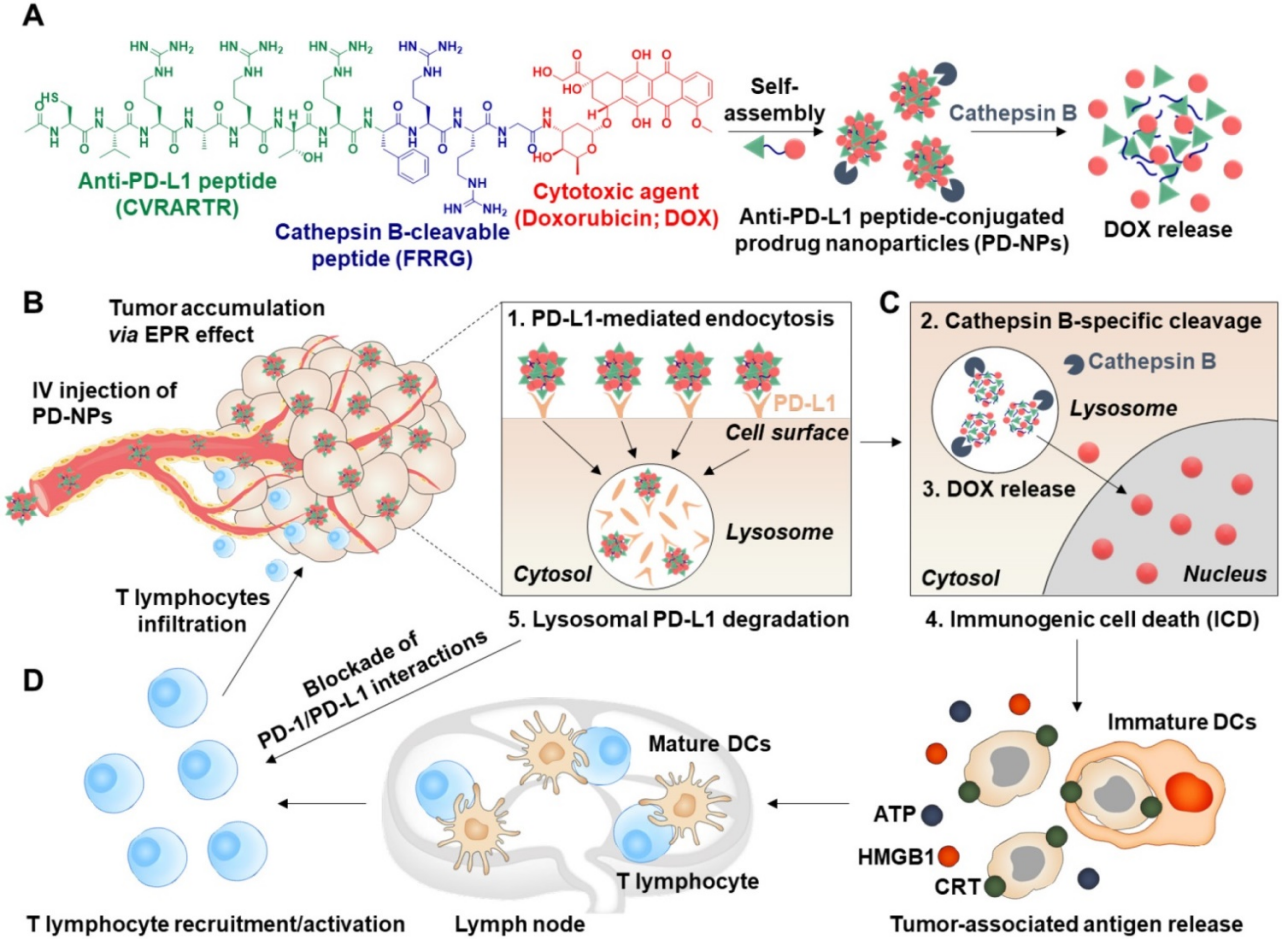
** Targeted cancer immunotherapy by PD-NPs to combine PD-L1 blockade with ICD. A.** PD-NPs are prepared by the self-assembly of anti-PD-L1 peptide (CVRARTR) and cathepsin B-specific cleavable peptide (FRRG), and doxorubicin (DOX) conjugates. **B.** The PD-NPs highly accumulate within targeted tumor tissues *via* EPR effect and then efficiently enter cancer cells through PD-L1 receptor-mediated endocytosis. **C.** Then, DOX molecules released from PD-NPs by cathepsin B overexpressed in the cancer cells induce effective ICD, which promote high DAMP signals for DC maturation and cytotoxic T lymphocyte activation. **D.** PD-NPs also disrupt the immune-suppressing PD-1/PD-L1 interactions *via* lysosomal PD-L1 degradation attributed from a potent binding affiinty to PD-L1, thereby enhancing pre-existing antitumor immune responses of T lymphocytes to destruct cancer cells.

Herein, we propose anti-PD-L1 peptide-conjugated prodrug nanoparticles (PD-NPs) that consist of anti-PD-L1 peptide (CVRARTR; *Cys-Val-Arg-Ala-Arg-Thr-Arg*), cathepsin B-specific cleavable peptide (FRRG; *Phe-Arg-Arg-Gly*) and doxorubicin (DOX) to combine ICD with ICB immunotherapy **(Scheme 1A)**. The functional peptide of CVRARTR*-*FRRG was directly conjugated to amine group of DOX *via* one-step amide bond reaction, and this direct conjugation of peptide and DOX can prevent non-specific drug release in off-target sites. This is because the target bioenzyme of cathepsin B overexpressed in cancer cells specifically cleave the -RRG- sequence in the PD-NPs to trigger DOX release in the targeted cancer cells, whereas the PD-NPs maintain non-toxic inactive state in normal cells with innately low cathepsin B expression 22-25. Therefore, PD-NPs can reduce severe inflammatory responses in normal organs and damage to the host immune system compared to conventional nanoparticles encapsulating drugs non-covalently that cause negative immunologic effects by premature drug leakage 8. The clinical outcomes of cancer immunotherapy rely importantly on the cross-talk between cancer cells and other cellular components, in particular immune cells in the tumor microenvironment 26. Thus, the PD-NPs can lead safe and more effective cancer immunotherapy by inducing ICD preferentially in targeted cancer cells and minimizing the off-target toxicity *via* cathepsin B-specific drug release. Importantly, amphiphilic CVRARTRFRRG-DOX conjugates spontaneously self-assembled into anti-PD-L1 peptide-conjugated prodrug nanoparticles (PD-NPs) *via* intermolecular interactions 27, 28. Therefore, PD-NPs achieve high and accurate anti-PD-L1 peptide and DOX loading contents (74.3 wt%) without any additional nano-sized carrier materials, which can solve the serious problems of conventional nanomedicines, such as low drug loading efficiency (<10 wt%), innate toxicity and difficulty in mass production. In tumor models, intravenously injected PD-NPs accumulate within targeted tumor tissues *via* enhanced permeability and retention (EPR) effect and then efficiently enter cancer cells through PD-L1 receptor-mediated endocytosis **(Scheme 1B)**. Then, the DOX molecules released from PD-NPs in the cathepsin B-overexpressed cancer cells induce ICD, which promote high DAMP signals for DC maturation and cytotoxic T lymphocyte activation **(Scheme 1C)**. In addition, PD-L1 binding of PD-NPs disrupt the immune-suppressing PD-1/PD-L1 interactions via lysosomal PD-L1 degradation, which enhance pre-existing antitumor immune responses of T lymphocytes to destruct cancer cells **(Scheme 1D)** 16. Concurrently, toxicity against normal cells and immune cells is greatly reduced by their innately low cathepsin B expression, thereby increasing the safety of ICB immunotherapy. In this study, we investigate the cancer immunotherapy, which induce a potent *in vivo* antitumor efficacy and immune responses with minimal side effects *via* combination of PD-L1 blockade with ICD by PD-NPs in breast tumor models.

## Methods

### Reagents

N-terminal acylated *Cys-Val-Arg-Ala-Arg-Thr-Arg-Phe-Arg-Arg-Gly* (Ac-CVRARTRFRRG-COOH) and *Cys-Val-Arg-Ala-Arg-Thr-Arg* (NH_2_-CVRARTR-COOH) peptides were synthesized from Peptron (Daejeon, Republic of Korea). Tem grid (Carbon Film 200 Mesh copper) was purchased from Electron Microscopy Sciences (Hatfield, PA, USA). Benzyloxycarbonyl-*Phe-Ala*-fluoromethylketone (Z-FA-FMK) and Monoclonal anti-mouse cathepsin B antibody were purchased from Santa Cruz Biotechnology (Dallas, Texas, USA). Cell counting kit-8 (CCK-8) was purchased from Vitascientific (Beltsville, MD, USA). CD8^+^ T cell enrichment column kit, recombinant cathepsin B, cathepsin D, cathepsin E, caspase-3, caspase-9 and TUNEL assays kit were purchased from R&D systems (Minneapolis, MN, USA). Doxorubicin hydrochloride (DOX) was purchased from Sigma Aldrich (Oakville, Ontario, USA). Dulbecco's Modified Eagle Medium (DMEM) high glucose medium, RPMI 1640 medium, fetal bovine serum (FBS), penicillin and streptomycin were purchased from WELGENE Inc. (Daegu, Republic of Korea). Anti-mouse calreticulin (CRT) antibody, Anti-mouse high mobility group box 1 (HMGB1) antibody, anti-β-actin antibody and IFN-γ ELISA Kit were purchased from Abcam (Hanam, Republic of Korea). Fluorescent dye-conjugated antibodies against mouse CD45.2, mouse CD8α, mouse CD3, mouse CD4, mouse CD25 and mouse CD86 (cat# 105005) were purchased from BioLegend (San Diego, CA, USA). Anti-mouse PD-L1 antibody (B7-H1) was purchased from BioXCell (Lebanon, NH, USA). 4T1 (human breast cancer cells) and H9C2 (rat BDIX heart myoblasts) cell lines were purchased from American Type Culture Collection (ATCC; Manassas, VA, USA). Tumor dissociation Kit and T Cell Activation/Expansion Kit were purchased from Miltenyi Biotechnology (Bergisch Gladbach, Germany). Six-week-old female Balb/c and Balb/c nu/nu mice were purchased from NaraBio, Inc (Seoul, Korea).

### Molecular dynamics simulation

The preliminary conformers of the molecules were searched by the MacroModel module in Schrödinger Suites (Schrödinger) with the following parameters-force field: OPLS3; solvent: water; charged from force field; cutoff: none; minimization method: PRCG; maximum iterations: 2,500; converge on: gradient; convergence threshold: 0.05; conformational search method: mixed torsional/low-mode sampling; torsional sampling options: intermediate; maximum number of steps:1000; energy window for saving structures: 21 kJ/mol; eliminate redundant conformers using maximum atom deviation cutoff: 0.5.

The molecular dynamics simulations were performed to investigate the packing mode of the two or twenty molecules via the Desmond module in Schrödinger Suites with the following parameters- force field: OPLS3e; solvent model: TIP4PD; ion placement: chloride; boundary conditions: orthorhombic box shape, box size calculation method (buffer); simulation time: 20 ns or 100 ns; approximate number of frames: 1000; ensemble class: NPT; temperature: 300 K; pressure: 1.01325 bar; thermostat method: Nose-Hoover chain; coulombic interaction cutoff radius: 9.0 Å. Intermolecular interactions were analyzed at every 10 frames.

### Cell preparation

4T1 (human breast cancer cells), HUVEC (human umbilical vein endothelial cells) and H9C2 (rat BDIX heart myoblasts) cells were maintained in RPMI-1640, Endothelial cell growth medium (EGM) or Dulbecco's Modified Eagle Medium (DMEM) supplemented with 10% (v/v) fetal bovine serum (FBS), 1% streptomycin and 100 U/mL penicillin, respectively. Bone marrow-derived macrophages (BMDMs) and bone marrow-derived dendritic cells (BMDCs) were prepared by isolating bone marrow cells from BALB/c mice. Then, bone marrow cells were differentiated into BMDMs by incubating with M-CSF (Macrophage colony-stimulating factor, 20 ng/mL) for 7 days, or were differentiated into BMDCs by culturing for 7 days in the presence of GM-CSF (20 ng/mL), IL4 (20 ng/mL) and 0.1% β-mercaptoethanol. After isolation, BMDMs and BMDCs were maintained in RPMI-1640 medium supplemented with 10% (v/v) FBS, 1% streptomycin, and 100 U/mL penicillin.

### Synthesis of anti-PD-L1 peptide-conjugated prodrug

The anti-PD-L1 peptide-conjugated prodrug was synthesized by one-step conjugation of N-terminal acylated *Cys-Val-Arg-Ala-Arg-Thr-Arg-Phe-Arg-Arg-Gly* peptide (Ac-CVRARTRFRRG-COOH) and doxorubicin (DOX) *via* EDC/NHS coupling chemistry. First, Ac-CVRARTRFRRG-COOH (1.8 g, 1.96 mmol), DOX (0.9 g, 1.66 mmol), NHS (400 mg, 3.48 mmol) and EDC (500 mg, 2.62 mmol) were dissolved in anhydrous DMF (100 mL) and then DIPEA (200 mg, 1.55 mmol) was subsequently added in mixture solution. After 6 h of incubation, prodrug was purified using high performance liquid chromatography (HPLC, Agilent Technologies, 1200 series, USA) and lyophilized at -90 ^o^C and 5 mTorr for 24 h to obtain as a red powder (Freeze Dryer, ilShinBioBase, Republic of Korea).

### Characterization of anti-PD-L1 peptide-conjugated prodrug nanoparticles

The purity and molecular weight were analyzed using HPLC and matrix-assisted laser desorption/ionization time of flight mass spectrometer (MALDI-TOF, AB Sciex TOF/TOF 5800 System, USA) with cyano-4-hydroycinnamic acid (CHCA) matrix, respectively. The average size of PD-NPs in saline (1 mg/mL) was determined *via* dynamic light scattering (DLS; Zetasizer Nano ZS Malvern Instruments, UK). The morphology of nanoparticles in distilled water (1 mg/mL) was observed by transmission electron microscope (TEM; CM-200, USA). As a control, the cathepsin B-cleaved PD-NPs were also observed using TEM, wherein PD-NPs (1mg/mL) were pre-incubated with cathepsin B (10 μg) at 37 ^o^C for 24 h. Enzyme cleavage assays were performed by incubating PD-NPs (10 μM) with cathepsin B, cathepsin D, cathepsin E, caspase-3 and caspase-9 (10 μg) in MES buffer (0.1 M, pH 5.5). As a control experiment, PD-NPs (10 μM) were also pre-incubated with irreversible cathepsin B inhibitor (Z-FA-FMK; 50 μΜ) in MES buffer and then further incubated with cathepsin B. Enzyme reaction buffer was analyzed by HPLC using an acetonitrile: H_2_O gradient from 20:80 (0 min) to 80:20 (20 min). *In vitro* drug release from the PD-NPs was further investigated under both physiological and enzymatic conditions. For these analyses, PD-NPs dispersed in the mouse serum (1 mg/mL) were loaded in membrane (Molecular weight cut off: 2000 Da; Dialysis membrane Spectra/Por^®^ 7 MWCO 2000, 18 mm; Carl Roth, Karlsruhe, Germany) in the presence or absence of cathepsin B enzyme (10 μg). After incubation, peptide-DOX or free DOX released to the outer membrane was analyzed using HPLC. Fluorescence profiles of PD-NPs (10 μM) in saline was analyzed by fluorescence spectrophotometer (F-7000, Hitachi).

### Cellular uptake

To assess the intracellular behavior of PD-NPs, 2 × 10^5^ 4T1, HUVEC and H9C2 cells were seeded in glass-bottom confocal dishes. After 24 h of stabilization, each cell was incubated with free DOX or PD-NPs (2 μM) at 37 °C. As a control, 4T1, HUVEC and H9C2 cells were pre-incubated with anti-PD-L1 antibody for 24 h to block the PD-L1 on cell surface. Then, cells were washed with DPBS, fixed with 4% paraformaldehyde for 10 min, and stained with 4',6-diamidino-2-phenylindole (DAPI) for 5 min. Fluorescence imaging was performed using a Leica TCS SP8 confocal laser-scanning microscope (CLSM; Leica Microsystems GmbH; Wetzlar, Germany).

### Cytotoxicity assay

The cytotoxicity of PD-NPs was assessed by cell counting kit-8 (CCK-8) assays. First, 1 x 10^4^ 4T1, HUVEC, H9C2, BMDMs, BMDCs or CD8^+^ T cells were seeded in 96-well cell culture plates. After 24 h of stabilization, the PD-NPs or free DOX were treated with each cell for 24 h. Then, the cells were further incubated with culture medium containing CCK-8 solution (10%) for 20 min, and cell viability was measured by a microplate reader with 450 nm of wavelength (VERSAmaxTM; Molecular Devices Corp., USA).

### Western blot

PD-L1 expression in 4T1 cells after different treatment was analyzed *via* western blot. Briefly, 1 × 10^6^ 4T1 cells were seeded in 6-well cell culture plates with RPMI medium containing 10% FBS and 1% antibiotics. After 24 h of stabilization, the cells were pre-treated with IFN-γ (20 ng/mL) for 24 h and further incubated with equal molar dose (2 μM) of free DOX, PD-NPs, anti-PD-L1 antibody or anti-PD-L1 peptide (NH_2_-CVRARTR-COOH) for 24 h. Then, each cell was solubilized with lysis buffer containing 1% protease inhibitors, and the lysates were centrifuged at 10,000 rpm for 15 min to remove cell debris. Proteins in lysates were quantified *via* BCA protein quantification kit (Thermo Fisher scientific, USA), and then separated by sodium dodecyl sulfate-polyacrylamide (SDS-PAGE) gel electrophoresis and transferred onto polyvinylidene difluoride (PVDF) membranes. Membranes were incubated with TBS-T containing 5% bovine serum albumin (BSA) for 50 min to block non-specific IgG binding and incubated with anti-mouse PD-L1 primary antibody (1000:1) for 12 h at 4 °C. Then, membranes were further incubated with HRP-conjugated anti-rabbit IgG antibody for 90 min at room temperature. Finally, immunoreactive bands were observed *via* enhanced chemiluminescence (ECL) system.

### DAMPs analysis

DAMPs from 4T1 cells were assessed by analyzing CRT, HMGB1 and ATP after treatment. First, 1 x 10^5^ 4T1 cells were incubated with free DOX or PD-NPs (2 μM) for 24 h. Then, cells were stained with a APC fluorescent dye-conjugated CRT antibody for 12 h at room temperature, followed by an analysis using a confocal laser-scanning microscope. Second, supernatants from each well were harvested to measure HMGB1 and ATP, which were analyzed *via* western blot and ATP assay kit, respectively (Beyotime Biotechnology). To evaluate the ICD of PD-NPs for DC maturation, 1 x 10^5^ 4T1 cells were cultured in 100-pi culture dishes. After 12 h of stabilization, cells were treated with free DOX or PD-NPs (2 μM) for 24 h. Then, cell culture medium containing DAMPs released from 4T1 cells was co-cultured with immature mouse bone marrow-derived dendritic cells (BMDCs) for 24 h, and the mature DCs were analyzed *via* flow cytometer (BD FACSVerse, BD Bioscience, USA) after staining with CD11c, CD40 and CD86.

### Co-culture of CD8+ T lymphocytes with cancer cells for analysis of T lymphocyte activity

CD8^+^ T cells were isolated from BALB/c mice using a CD8 enrichment column and then activated by culturing on plates coated with anti-mouse CD3 antibody (1 μg/mL) in culture medium containing anti-mouse CD28 antibody at 37 ^o^C for 3 h. Activated T lymphocytes were co-cultured with 4T1 cells pre-treated with anti-PD-L1 peptide, anti-PD-L1 antibody or PD-NPs (2 μM) at 37 ^o^C for 24 h. To analyze T lymphocyte activity, the secretion of IFN-γ and T lymphocyte proliferation were measured. The levels of IFN-γ in the cell culture medium was measured using a IFN-γ ELISA Kit (Hanam, Republic of Korea) and T lymphocyte proliferation was measured by flow cytometry as below.

CD8^+^ T lymphocytes were labeled with CellTrace^TM^ Far Red dye (Thermo Fisher Scientific Inc., Rockford, IL, USA) resuspended in dimethyl sulfoxide at a concentration of 5 mM, and the dilution assays of CellTrace^TM^ Far Red dye was performed to analyze T lymphocyte proliferation. Briefly, activated T lymphocytes were incubated with Far Red dye in serum-free culture medium at 37 ^o^C for 10 min, and then cells were resuspended in RPMI1640 medium containing 10% FBS. After 24 h of co-culture with 4T1 cells pre-treated with anti-PD-L1 peptide, anti-PD-L1 antibody or PD-NPs, the decreased fluorescence intensities of daughter T lymphocytes was compared to parental cells after cell division due to the dilution of Far Red dye.

### Biodistribution study in breast tumor models

The 6-week female BALB/c mice were purchased from NaraBio (Gyeonggi-do, Republic of Korea). Mice were bred under pathogen-free conditions in the Korea Institute of Science and Technology (KIST). All experiments with animals were performed in compliance with the relevant laws and institutional guidelines of Institutional Animal Care and Use Committee (IACUC; approved number of 2020-123) in Korea Institute of Science and Technology (KIST). The *in vivo* biodistribution of PD-NPs was assessed in 4T1 tumor-bearing mice, which were prepared by subcutaneous inoculation of 1 x 10^7^ 4T1 cells. When the tumor volumes were approximately 150 - 200 mm^3^, the mice were intravenously injected with free DOX or PD-NPs (3 mg/kg based on DOX contents). Noninvasive near-infrared fluorescence (NIRF) imaging was performed using an IVIS Lumina Series III system (PerkinElmer; Waltham, MA, USA) after 1, 6 and 24 h of injection. Fluorescence intensity in tumor regions was quantified using a Living Image software (PerkinElmer; Waltham, MA, USA). For *ex vivo* fluorescence imaging, mice were sacrificed after 24 h of injection, followed by extraction of liver, lung, spleen, kidney, heart and tumor tissues. Fluorescence intensity in tissues was analyzed using an IVIS Lumina Series III system. Tumor tissues were cut into 8-μm thick sections for histological assays. Slide-mounted tumor sections were stained with APC fluorescent dye-conjugated CD31 or PD-L1 antibody at 4 ^o^C for 12 h, after which tumor tissues were analyzed using a Leica TCS SP8 confocal laser-scanning microscope.

### *In vivo* tumor inhibition and immune response

4T1 tumor-bearing mice were randomly divided into four groups: (i) saline; (ii) free DOX; (iii) free DOX with anti-PD-L1 antibody; and (iv) PD-NPs. Then, mice were treated once every three days, with free DOX or PD-NPs (3 mg/kg based on DOX contents), at which time tumor volumes had reached 60 - 80 mm^3^. The anti-PD-L1 antibody (10 mg/kg) was i.p. injected simultaneously with free DOX into mice. Antitumor efficacy was assessed by measuring tumor volumes, calculated as largest diameter x smallest diameter^2^ x 0.53, every 2 days. For the analysis of antitumor immune responses, tumor tissues were collected on day 7, and single cells were isolated from tumor tissues using a Tumor Dissociation Kit. Following cell counting, the single cells were incubated with FcBlock at 37 ^o^C for 5 min to avoid non-specific antibody binding. Then, multi-parameter staining was performed for 40 min to assess the following populations in tumor tissues; (i) PD-L1^+^ cancer cells (CD45^-^PD-L1^+^); (ii) CRT^+^ cancer cells (CD45^-^CRT^+^); (iii) CD8^+^ T cells (CD45^+^CD3^+^CD8^+^); and (iv) regulatory T cells (Tregs; CD45^+^CD3^+^CD4^+^CD25^+^). In order to assay the concentration of cytokines in the tumor microenvironment, the IFN-γ and TNF-α in the tumor supernatants were assessed using commercialized ELISA assay kits (Catalog # SMIF00 and SMTA00B, respectively, R&D systems).

### Toxicity study

The *in vivo* toxicity of PD-NPs was assessed by hematological analyses. Briefly, free DOX or PD-NPs (3 mg/kg based on DOX contents) were intravenously injected once every three days, and anti-PD-L1 antibody (10 mg/kg) was simultaneously i.p. injected into mice. Then, blood samples were collected from mice on day 16. For complete blood counts (CBCs), each blood sample was mixed with EDTA and a portion of each blood sample was centrifuged at 1,000 xg for 20 min, and then supernatants were stored at 4 °C. The following factors in blood samples were measured; aspartate transaminase (AST), alanine aminotransferase (ALT), blood urea nitrogen (BUN), creatinine kinase-MB (CK-MB), white blood cell (WBC), neutrophil (NEUT) and lymphocytes (LYMP).

### Statistics

The statistical significance between two groups was analyzed using Student's t-test. One-way analysis of variance (ANOVA) was performed for comparisons of more than two groups, and multiple comparisons were analyzed using Tukey-Kramer *post-hoc* test. Survival data was plotted as Kaplan-Meier curves and analyzed using log-rank test. Statistical significance was indicated with asterisk (*P < 0.05, **P < 0.01, ***P < 0.001) in the figures.

### Data availability

All relevant data are available with the article and its Supplementary Information files, or available from the corresponding authors upon reasonable request.

## Results and Discussion

### Characterization of anti-PD-L1 peptide-conjugated prodrug nanoparticles (PD-NPs)

**Figure 1:**
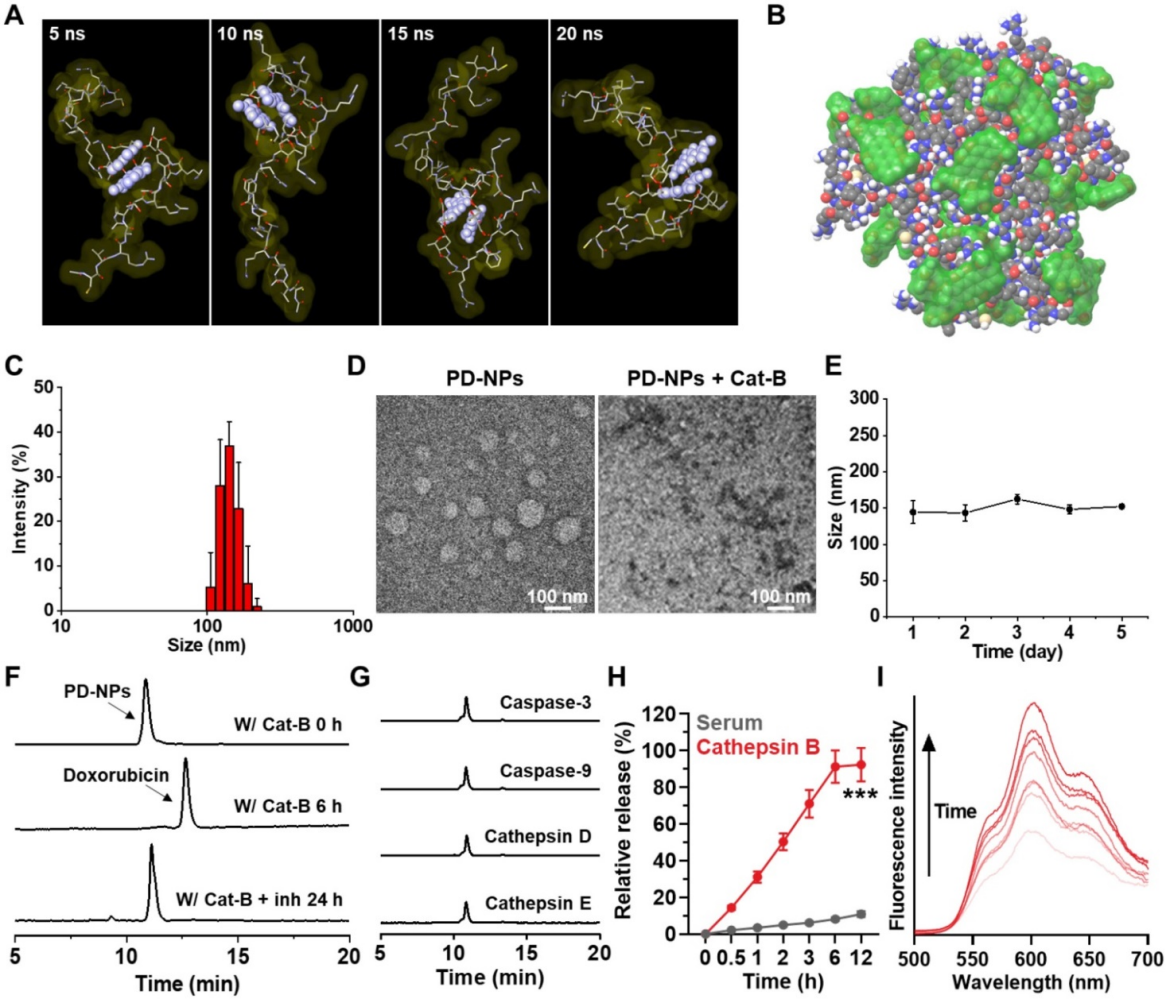
** Physicochemical characterization of PD-NPs. A.** All atom molecular dynamics (MD) simulation results of two molecules of peptide-drug conjugates. Aromatic segment of DOX was highlighted by light purple, for clarity. **B.** MD simulation of twenty molecules of peptide-drug conjugates. Molecular surfaces of DOX were colored by translucent green. **C.** Size distribution of PD-NPs in saline was confirmed using dynamic light scattering (DLS). **D.** TEM image of PD-NPs in distilled water. As a control, PD-NPs incubated with cathepsin B (10 μg) at 37 ^o^C for 24 h were observed *via* TEM. **E.** Size stability of PD-NPs in mouse serum for 5 days. **F-G.** HPLC spectrum when the PD-NPs were incubated with **F.** cathepsin B (pH 5.5) or cathepsin B with inhibitor (Z-FA-FMK), and **G.** other enzymes. **H.** *In vitro* drug release from PD-NPs in serum in the presence or absence of cathepsin B enzyme. **I.** Time-dependent fluorescence spectra of PD-NPs in the presence of cathepsin B for 48 h. Y-axis indicates the fluorescence intensity from PD-NPs. The DOX fluorescence (590 nm) from PD-NPs was gradually increased in a time-dependent manner after incubation with cathepsin B owing to de-quenching effect by enzymatic degradation. Significance was determined by Student's *t* test (H).

The anti-PD-L1 peptide-conjugated prodrug nanoparticles (PD-NPs) that consist of anti-PD-L1 peptide (CVRARTR; *Cys-Val-Arg-Ala-Arg-Thr-Arg*), cathepsin B-specific cleavable peptide (FRRG; *Phe-Arg-Arg-Gly*) and doxorubicin (DOX) were designed for targeted cancer immunotherapy combining PD-L1 blockade with ICD. The anti-PD-L1 peptide of CVRARTR had a potent binding affinity (KD) of 281 ± 92 nM, and it was further modified with cathepsin B-specfic cleavable peptide of FRRG 13. It was also reported that -RRG- sequence in FRRG peptide was recognized and cleaved by cathepsin B enzyme 22. To prepare anti-PD-L1 peptide-conjugated prodrug, N-terminal acylated functional peptide (Ac-CVRARTRFRRG-COOH) was directly conjugated to amine group of DOX *via* a one-step EDC/NHS reaction **(Figure S1).** After the reaction, 99% of peptide-DOX conjugates was purified with HPLC **(Figure S2A)**. The molecular weight of peptide-DOX conjugate was calculated to be 1945.19 Da for C_85_H_129_N_27_O_24_S, and measured to be 1945.8717 m/z [M] *via* MALDI-TOF mass spectrometer **(Figure S2B)**. Importantly, the amphiphilic peptide-DOX conjugates spontaneously self-assembled into nanoparticles in aqueous condition *via* intermolecular interactions 27, 28. Therefore, the resulting PD-NPs exhibited a high drug loading contents of 74.3 wt% including 46.4% of anti-PD-L1 peptide (CVRARTR; 903.08 m/z) and 27.9% of DOX (543.53 m/z) in the peptide-DOX conjugates (1945.8717 m/z). The molecular dynamics (MD) simulation results of two molecules of peptide-DOX conjugates indicated that aromatic segments of DOX maintain the average distance of 5.5 Å for 20 ns, which demonstrate that driving force to promote the self-assembly of peptide-drug conjugates is aromatic interactions of DOX molecules **(Figure 1A and S3)**. In addition, all atom MD simulation of twenty molecules of peptide-DOX conjugates further showed a micellar aggregate *via* π-π stacking, π-cation interactions, and hydrogen bonds, respectively **(Figure 1B and S4)**. The PD-NPs showed an average size of 157.4 ± 12.1 nm in saline, as confirmed by dynamic light scattering **(**DLS; **Figure 1C)**. TEM images also indicated the spherical structure of PD-NPs, and their nano-sized structure was dissociated after incubation with cathepsin B at 37 ^o^C for 24 h **(Figure 1D)**. In addition, PD-NPs were well-dispersed in mouse serum without significant change in particle size for 5 days **(Figure 1E)**. These stable nanoparticle characteristics of PD-NPs in blood are expected that PD-NPs can increase tumor accumulation *via* EPR effect *in vivo* 29. The target enzyme-specific cleavage of PD-NPs was assessed after incubation with various enzymes. When PD-NPs were incubated with cathepsin B (10 μg) in MES buffer (pH 5.5), nanoparticles began to release free DOX after 1 h of incubation, and approximately 99% was cleaved at 6 h post-incubation **(Figure 1F)**. This was clearly supported by MALDI-TOF result that confirm molecular weights of free DOX (calculated mass: 543.52 Da, measured mass: 543.009 m/z [M], 549.947 m/z [M+Li], 567.9522 m/z [M+Na]) at a newly apperared characteristic peak (12 min) after incubation with cathepsin B enzyme in HPLC spectrum **(Figure S5)**. In contrast, PD-NPs were not cleaved in the cathepsin B-suppressed condition that cathepsin B is pre-incubated with irreversible cathepsin B inhibitor **(Figure 1F)**. Similar results were also observed when PD-NPs were incubated with other enzymes, such as caspase-3, caspase-9, cathepsin D and cathepsin E for 24 h of incubation **(Figure 1G)**. The *in vitro* drug release of PD-NPs under physiological and enzymatic conditions was further investigated, wherein the PD-NPs dispersed in mouse serum (1 mg/mL) were loaded in the membranes (Molecular weight cut off: 2000 Da) in the presense or absence of cathepsin B enzyme. The results showed that only approximately 10% of peptide-DOX was released to the outer membrane, but over 90% of free DOX was observed in same condition in the presence of cathepsin B, indicating high target enzyme-specificity and stability in physiological condition **(Figure 1H)**. As a control, the cathepsin B-scrambled functional peptide-conjugated DOX (CVRARTR-FGRG-DOX) was not cleaved by cathepsin B, indicating high target enzyme specificity of -RRG- peptide sequence **(Figure S6)**. Finally, the fluorescence intensities of PD-NPs in saline were gradually increased in a time-dependent manner after incubation with cathepsin B **(Figure 1I)**. These results demonstrate that PD-NPs maintain self-quenched inactive state as a stable nano-sized structure, but release free DOX by enzymatic degradation in the presence of cathepsin B.

### PD-L1 binding and cancer cell-specific cytotoxicity of PD-NPs

**Figure 2:**
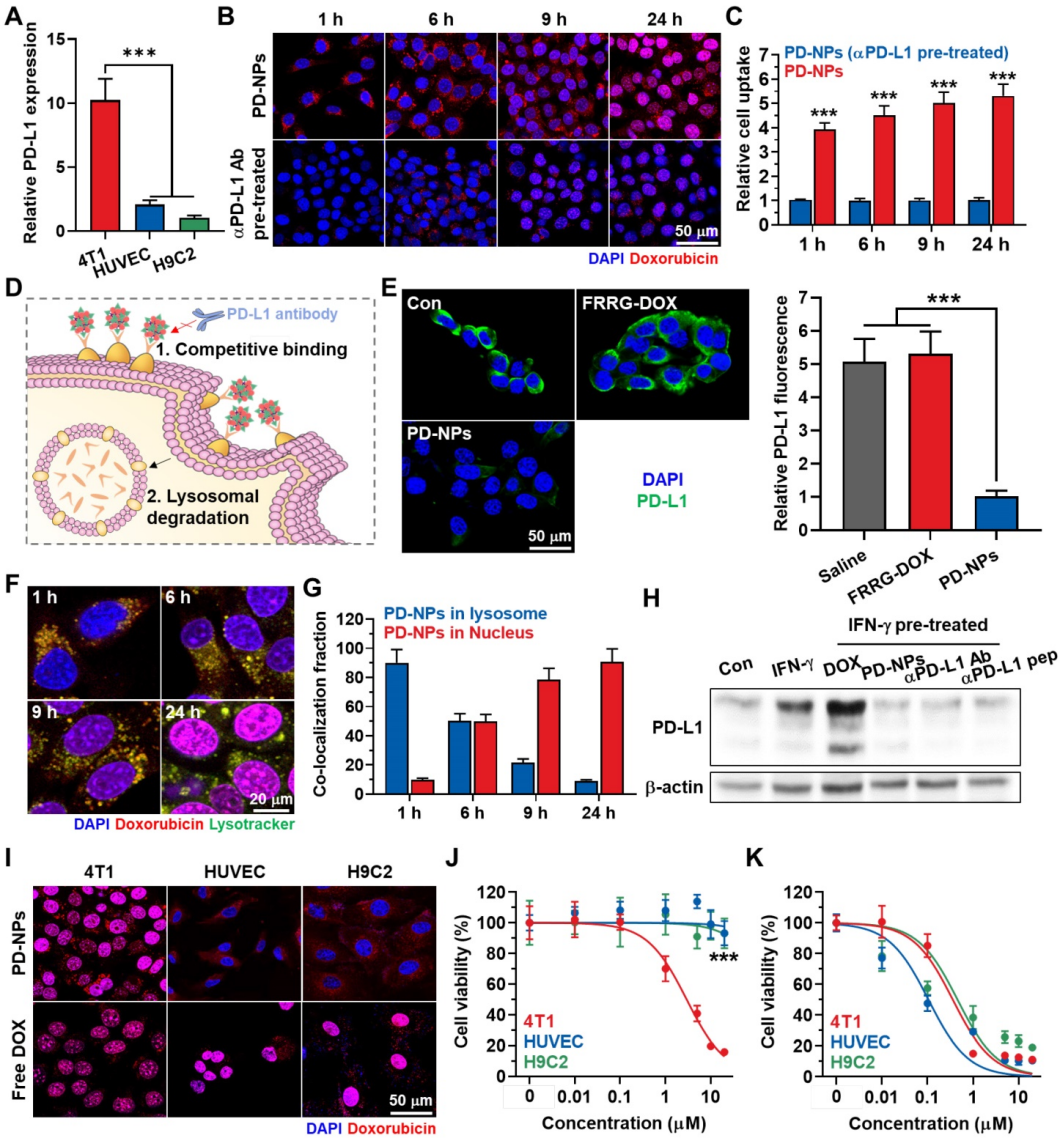
** PD-L1 binding and cancer cell-specific cytotoxicity of PD-NPs. A.** Relative PD-L1 expression in 4T1, HUVEC and H9C2 cells. **B.** Time-dependent cellular uptake of PD-NPs in 4T1 cells. As a control, anti-PD-L1 antibody was pre-incubated with 4T1 cells for 24 h before PD-NPs treatment. **C.** Quantification analysis on the amount of cellular uptake of PD-NPs in 4T1 or anti-PD-L1 antibody-treated 4T1 cells. **D.** Schematic illustration to explain the mechanism of PD-L1 blockade by PD-NPs. **E.** Fluorescence imaging of APC-conjugated anti-PD-L1 antibody-treated 4T1 cells after pretreatment of FRRG-DOX or PD-NPs for 2 h at 4 ^o^C. **F.** Lysosome co-localization of PD-NPs in 4T1 cells. Intracellular lysosomes were labeled with Lamp1-RFP. **G.** Quantitative analysis of the co-localization of PD-NPs with lysosomes (Lamp1-RFP) or nuclei (DAPI) in the 4T1 cells. **H.** PD-L1 expression on the cell surface after different treatment was analyzed *via* western blot. **I.** Fluorescence imaging of 4T1, HUVEC and H9C2 cells treated with PD-NPs or free DOX for 24 h. **J-K.** Cytotoxicity of **J.** PD-NPs and **K.** free DOX in 4T1, HUVEC and H9C2 cells after 24 h treatment. Significance was determined by Student's *t* test (C) or Tukey-Kramer *posthoc* test (A, E, J).

The *in vitro* binding between PD-NPs and PD-L1 on the cell surface was assessed in breast cancer cells. This is because breast cancer express significantly higher levels of PD-L1 compared to other cancers in clinic 30. Moreover, breast cancer cells (4T1) expressed 5.01 ± 0.48-fold and 10.23 ± 1.66-fold high levels of PD-L1 in comparison to normal cells of human umbilical vein endothelial cells (HUVEC) and rat BDIX cardiomyocytes **(**H9C2; **Figure 2A and S7)**. In cultured condition, PD-NPs (red color) were efficiently taken up by 4T1 cells in an incubation time-dependent manner **(Figure 2B)**. However, pretreatment with anti-PD-L1 antibody to block the PD-L1 on cell surface significantly reduced the cellular uptake of PD-NPs, indicating that PD-NPs enter cancer cells through PD-L1 receptor-mediated endocytosis **(Figure 2B)**. Quantitatively, cellular uptake of PD-NPs was 5.314 ± 0.481-fold higher in naive 4T1 cells than in anti-PD-L1 antibody-pretreated 4T1 cells after 24 h of incubation **(Figure 2C)**. Moreover, these fluorescence imaging also showed that intracellular PD-NPs remained to the cytosol until 6 h of incubation, whereas DOX fluorescence was clearly observed in the nuclei from 9 h of incubation. Upon enhanced internalization driven by PD-L1-binding, DOX delivery into the nuclei was 3.04 ± 0.28-fold higher in naive 4T1 cells than in anti-PD-L1 antibody-pretreated 4T1 cells **(Figure S8)**. The mode of action (MOA) of DOX is intercalating within DNA base pairs to cause breakage of DNA strand and inhibition of both RNA and DNA replication. Therefore, DOX inside the nuclei of the cells is an important indicator for its potent cytotoxicity. As control experiments, the non-specific binding of PD-NPs on HUVEC was evaluated because the PD-L1 expression levels of HUVEC cells are significantly higher than other normal cells of H9C2. However, the low cellular binding and uptake of PD-NPs were observed in HUVEC and H9C2, and those were similar irrespective of the pretreatment of anti PD-L1 antibody owing to much low PD-L1 expression than 4T1 cancer cells **(Figure S9)**.

Next, we further assessed whether the binding to PD-L1 of PD-NPs can lead to blockade of PD-L1 *via* competitive binding and lysosomal PD-L1 degradation, and detail mechanisms were shown in **Figure 2D**. First, pretreatment with PD-NPs for 2 h at 4 ^o^C significantly reduced APC-conjugated anti-PD-L1 antibody binding to PD-L1 on the cell surface *via* competitive binding, whereas pretreatment of FRRG-DOX that absence of anti-PD-L1 peptide (CVRARTR) did not affect to the PD-L1 antibody binding compared to naive cells **(Figure 2E)**. We have also demonstrated substantial PD-NPs internalized into lysosomes, wherein the strong co-localization of PD-NPs (red color) and lysosome (green color) was clearly observed in 4T1 cells **(Figure 2F)**. After 1 h of incubation, approximately 90% of PD-NPs was localized in the lysosomes, whereas DOX fluorescence in nuclei was gradually increased from 6 h post-incubation **(Figure 2G)**. Recent study noted that controlled trafficking of PD-L1 to targeted degradation in the lysosome might be a key to prevent unwanted PD-L1 recycling, thus these intracellular fate of PD-NPs can also increase the lysosomal PD-L1 degradation 16. As a result, the treatment of 4T1 cells with IFN-γ for 24 h resulted in significant PD-L1 upregulation, and subsequent treatment of free DOX further increased the PD-L1 expression **(Figure 2H)**. However, PD-NPs significantly downregulated the PD-L1 expression levels of IFN-γ-treated 4T1 cells, and their treatment-induced downregulation of PD-L1 was nearly similar with anti-PD-L1 peptide (CVRARTR) or antibody. These results clearly indicate that PD-NPs successfully block the recognition of PD-L1 on cell surface by anti-PD-L1 antibody that can be considered a PD-1 of T lymphocytes *via* competitive binding and lysosomal degradation for PD-L1.

The cancer cell-specific cytotoxicity of PD-NPs premised on differential cathepsin B expression was assessed in 4T1, HUVEC and H9C2 cells. As expected, 4T1 cells expressed 26.13 ± 2.78-fold and 32.48 ± 3.14-fold of cathepsin B, and 4.39 ± 0.41-fold and 5.62 ± 0.581-fold of pro-cathepsin B compared to HUVEC and H9C2 cells, respectively **(Figure S10)**. Three types of cells showed robust uptake of PD-NPs (red color) after 24 h of incubation as confirmed by confocal laser scanning microscope **(**CLSM; **Figure 2I)**. Importantly, DOX fluorescence of PD-NPs remained limited to the cytoplasm of HUVEC and H9C2 cells, whereas that was observed in the nuclei of the 4T1 cells. In contrast, intracellular behavior of free DOX was similar regardless of cathepsin B expression in cells, wherein DOX fluorescence was clearly detected in the nuclei of three types of cells. Furthermore, such different intracellular behavior of PD-NPs resulted in cancer cell-specific cytotoxicity. The IC_50_ values of PD-NPs were measured to be 3.01 μM, 200 μM and > 200 μM in 4T1, HUVEC and H9C2 cells, respectively, which showed over a 70-fold difference that indicate greatly minimized cytotoxicity against normal cells **(Figure 2J)**. In contrast, free DOX exhibited indiscriminate cytotoxicity with similar IC_50_ in three types of cells **(Figure 2K)**. Taken together, these results indicate that PD-NPs specifically release free DOX into the nuclei of cathepsin B-overexpressed cancer cells, whereas maintain non-toxic inactive state in the cytosol of normal cells with innately low cathepsin B expression. In addition, these cancer cell-specific cytotoxicity of PD-NPs also suggest that PD-NPs can greatly mitigate the side effects of ICB immunotherapy by minimized toxicity towards normal tissues.

### *In vitro* immunogenic cell death and reinvigoration of T lymphocytes by PD-NPs

**Figure 3:**
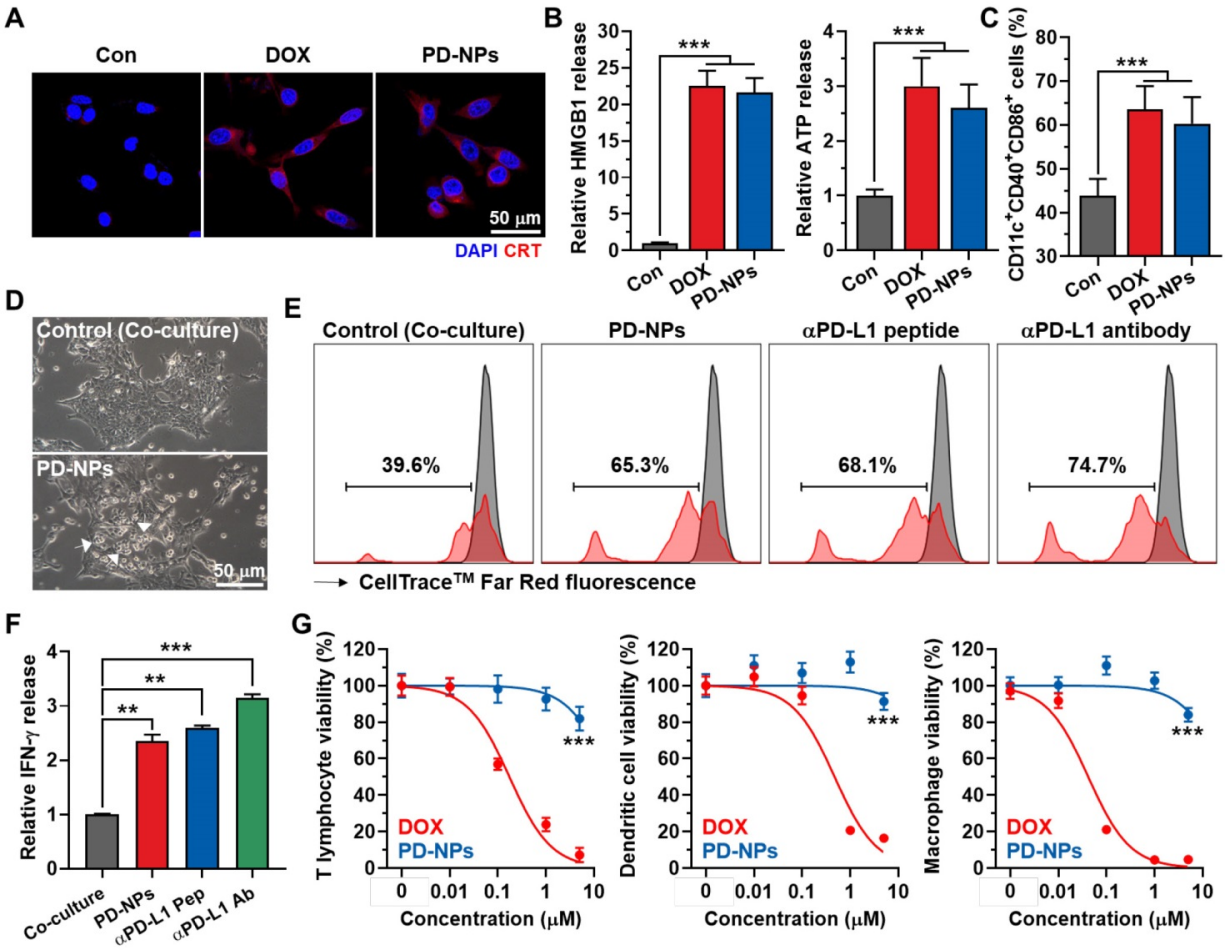
** ICD and reinvigoration of T lymphocytes by PD-NPs. A.** CRT expression in the 4T1 cells after saline, free DOX or PD-NPs treatment. **B.** Quantification analysis on the amount of HMGB1 and ATP released from 4T1 cells after saline, free DOX or PD-NPs treatment. **C.** The levels of mature DCs (CD11c^+^CD40^+^CD86^+^) in BMDCs after co-culture with culture medium containing DAMPs released from 4T1 cells treated with free DOX or PD-NPs (2 μM). **D.** Photographs of T lymphocytes co-cultured with naive or PD-NPs-treated 4T1 cells for 24 h. **E.** T lymphocyte proliferation assays after 24 h of co-culture with 4T1 cells treated with PD-NPs, anti-PD-L1 peptide (CVRARTR) or anti-PD-L1 antibody. **F.** Relative amount of IFN-γ in culture medium after co-culture of T lymphocyte with 4T1 cells treated with PD-NPs, anti-PD-L1 peptide (CVRARTR) or anti-PD-L1 antibody for 24 h. **G.** Cytotoxicity of PD-NPs and free DOX in T lymphocytes, DCs and macrophages after 24 h treatment. Significance was determined by Student's *t* test (G) or one-way ANOVA with the Tukey-Kramer *posthoc* test (B, C, F).

The immunogenic cell death (ICD) by PD-NPs in cancer cells was examined by measuring the amount of DAMPs, such as caleticulin (CRT) expression on the cell surface, extracellular release of high mobility group box 1 (HMGB1) and adenosine triphosphate (ATP). After 24 h of treatment, the CRT expression on the surface of 4T1 cells treated with PD-NPs was similar with free DOX **(Figure 3A)**. In addition, there was no significant differences in extracellular release of HMGB1 and ATP from 4T1 cells treated with PD-NPs or free DOX, and those were significantly higher than naive cells **(Figure 3B and S11)**. The effective ICD by PD-NPs was further evaluated in co-culture study. For these analyses, 4T1 cells were treated with free DOX or PD-NPs (2 μM) for 24 h, and cell culture medium containing DAMPs released from the cells was incubated with immature mouse bone marrow-derived dendritic cells (BMDCs) for 24 h. The results showed the equivalent levels of mature DCs (CD11c^+^CD40^+^CD86^+^) in BMDCs after each drug treatment, which is attributable to the DAMPs induced by strong ICD of the PD-NPs **(Figure 3C and S12)**. To examine the ability of PD-NPs to reinvigorate T lymphocyte activity by blockade of interactions between PD-1 and PD-L1, we exploited the co-culture of T lymphocytes with 4T1 cells. For this study, CD8^+^ T lymphocytes were isolated from mice spleen and activated by culturing with medium containing anti-CD28 antibody on anti-CD3 antibody-coated plates, and subsequently co-cultured with 4T1 cells for 24 h. Interestingly, T lymphocytes located near 4T1 cells treated with PD-NPs was considerably increased than naive 4T1 cells after washout, suggesting the increase of cancer cell recognition by T lymphocytes **(Figure 3D)**. As a result, co-culture with PD-NPs-treated 4T1 cells significantly increased the T lymphocyte proliferation and the secretion of IFN-γ from T lymphocytes (2.35 ± 0.13-fold compared to control), and those were similar with anti-PD-L1 peptide (CVRARTR) and antibody groups **(Figure 3E-F)**. These results indicate that PD-NPs successfully block the interaction between PD-1 and PD-L1 to enhance T lymphocyte target recognition, which lead reinvigoration effect of T lymphocytes. We further assessed the cytotoxicity of PD-NPs against immune cells, such as T lymphocytes, dendritic cells (DCs) and macrophages. Because it has reported that immune cells express relatively lower cathepsin B compared to cancer cells, we expected that PD-NPs can reduce cytotoxicity towards immune cells to allerviate their dysfunction 5. The results showed that cytotoxicity of PD-NPs was significantly reduced in T lymphocytes (P < 0.001), DCs (P < 0.001) and macrophages (P < 0.001) compared to free DOX **(Figure 3G)**. Taken together, PD-NPs can elicit effective ICD in cancer cells by DOX release as well as promote proliferation and reinvigoration of T lymphocytes by PD-L1 blockade. In addition, PD-NPs based ICB immunotherapy may greatly reduce damage to host immune system owing to minimized toxicity against immune cells.

### Antitumor efficacy and immune reponses by PD-NPs in breast tumor models

**Figure 4:**
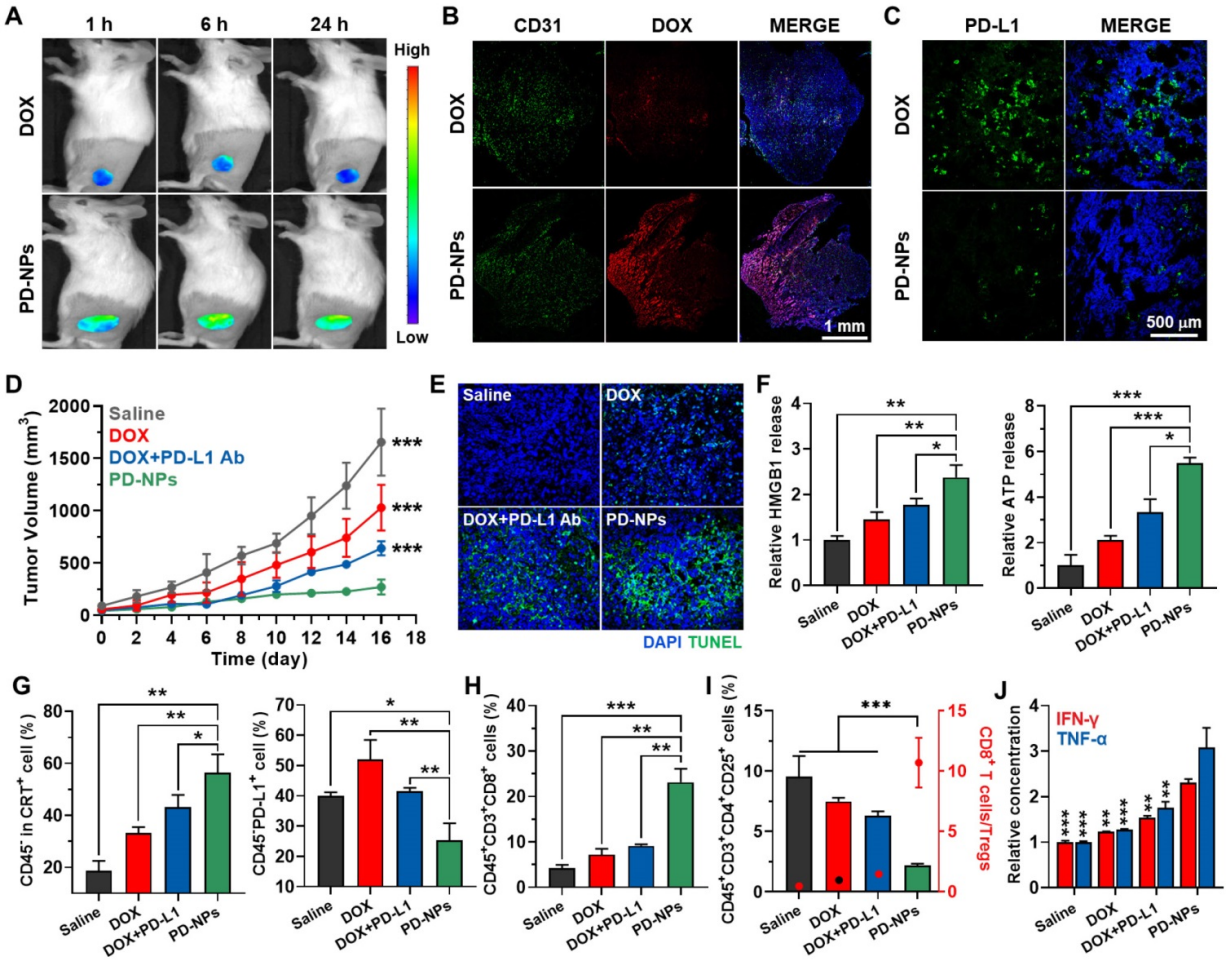
** Antitumor efficacy and immune responses by PD-NPs. A.** Noninvasive NIRF images of 4T1 tumor-bearing mice treated with free DOX or PD-NPs (3 mg/kg based on DOX contents). **B.** Tumor tissues stained with APC-conjugated CD31 antibody after 24 h of free DOX or PD-NPs treatment. **C.** Tumor tissues stained with APC-conjugated PD-L1 antibody after 24 h of free DOX or PD-NPs treatment. **D.** Tumor growth curves of 4T1 tumor-bearing mice during saline, free DOX, free DOX with PD-L1 antibody, or PD-NPs treatment. Mice were treated once every three days. **E.** Tumor tissues stained with TUNEL to assess apoptosis on day 16 after treatment. **F.** Relative amount of HMGB1 and ATP in the tumor supernatants were analyzed on day 7 after treatment. **G-I.** Percentage of **G.** CRT-positive cancer cells (CD45^-^CRT^+^), PD-L1-positive cancer cells (CD45^-^PD-L1^+^), **H.** cytotoxic T lymphocytes (CD45^+^CD3^+^CD8^+^), **I.** Tregs (CD45^+^CD3^+^CD4^+^CD25^+^) and the ratio of CD8^+^ T lymphocytes to Tregs in the tumor tissues on day 7 after treatment. **J.** Relative concentration of the IFN-γ and TNF-α in the tumor supernatants from 4T1 tumor-bearing mice after 7 days of treatment. The asterisks in Figure indicate the significance in comparison to PD-NPs group. Significance was determined by one-way ANOVA with the Tukey-Kramer *posthoc* test.

The tumor-targeting ability of PD-NPs was monitored in breast tumor models *via* noninvasive near-infrared fluorescence (NIRF) imaging. The 4T1 tumor-bearing mice were prepared by subcutaneous inoculation of 1 x 10^6^ 4T1 cells into the BALB/c mice. When the tumor volumes were approximately 150 - 200 mm^3^, PD-NPs or free DOX (3 mg/kg based on DOX contents) were intravenously injected into the mice. The NIRF intensity of free DOX is not large enough *in vivo* with limited tissue penetration to screen the fluorescence of PD-NPs in whole body, thus fluorescence imaging was performed by appointing the tumor areas. In case of free DOX, only weak DOX fluorescence was observed at targeted tumor tissues and then rapidly decreased after 6 h of injection **(Figure 4A)**. In contrast, PD-NPs at tumor tissues were gradually increased until 6 h post-injection and sustainably retained for 24 h, which showed 4.01-4.48 times more accumulation compared to free DOX after 24 h injection **(Figure S13)**. These NIRF imaging confirm efficient tumor accumulation of PD-NPs *via* nanoparticle-derived EPR effect. The histological assay of whole tumor tissues stained with CD31 also showed high tumor accumulation of PD-NPs than free DOX, wherein the strong DOX fluorescence of PD-NPs (red color) along to CD31-positive blood vessels (green color) was clearly observed **(Figure 4B)**. In addition, tumor tissues stained with APC-conjugated anti-PD-L1 antibody showed low PD-L1 expression in PD-NPs group compared to free DOX group with PD-L1 upregulation, indicating the successful PD-L1 blockade by PD-NPs **(Figure 4C)**.

Next, the enhanced antitumor efficacy and immune responses by PD-NPs were assessed in 4T1 tumor-bearing mice. The mice were randomly divided into four groups of saline, free DOX (3 mg/kg), free DOX with anti-PD-L1 antibody (10 mg/kg), and PD-NPs (3 mg/kg based on DOX contents). Each drug was intravenously injected into mice once every three days, and anti-PD-L1 antibody was i.p. injected simultaneously with free DOX. Importantly, the PD-NPs group (271.15 ± 71.49 mm^3^) showed minimal tumor progression on day 16 compared to saline (1655.9 ± 319.94 mm^3^), free DOX (1028.73 ± 218.15 mm^3^), and free DOX with anti-PD-L1 antibody **(**641.45 ± 67.22 mm^3^) groups **(Figure 4D)**. Tumor tissues stained with TUNEL clearly exhibited that PD-NPs treatment resulted in elevated apoptosis than other treatments, thereby confirming the enhanced antitumor efficacy by targeted tumor delivery of DOX and anti-PD-L1 peptide **(Figure 4E)**. Next, we investigated the antitumor immune responses by measuring DAMPs from tumor tissues on day 7 after treatment. Firstly, extracellular release of HMGB1 and ATP into the tumor supernatants was significantly increased in the PD-NPs group than other groups **(Figure 4F and S14)**. In addition, the population of CRT positive tumor cells, PD-L1 positive tumor cells, cytotoxic T lymphocytes and regulatory T lymphocytes in tumor tissues was analyzed by flow cytometry on day 7 after treatment **(Figure S15)**. In case of free DOX, the population of CRT positive tumor cells (CD45^-^CRT^+^) was increased by inducing ICD, but PD-L1 positive tumor cells (CD45^-^PD-L1^+^) were also increased compared to saline group **(Figure 4G)**. In contrast, free DOX with anti-PD-L1 antibody group showed significantly increased CRT positive tumor cells, whereas PD-L1 positive tumor cells were decreased compared to free DOX group. Most importantly, PD-NPs group showed a most high population of CRT positive tumor cells (CD45^-^CRT^+^), while PD-L1 positive tumor cells (CD45^-^PD-L1^+^) were dramatically decreased compared to other groups. These results indicate the effective ICD and PD-L1 blockade by PD-NPs, which induced immune-responsive tumors by recruiting a large amount of immune cells at the tumor tissues. Consequentially, the population of cytotoxic T lymphocytes (CD45^+^CD3^+^CD8^+^) in the tumor tissues was considerably increased in the PD-NPs group **(Figure 4H)**. In contrast, the percentage of regulatory T cells (Tregs; CD45^+^CD3^+^CD4^+^CD25^+^) in the tumor tissues was significantly decreased in the PD-NPs group, wherein the ratio of CD8^+^ T lymphocytes to Tregs was greatly upregulated compared to other groups **(Figure 4I)**. Finally, the IFN-γ and TNF-α in the tumor microenvironment were significantly increased in the PD-NPs group, indicating high activity of tumor-infiltrating lymphocytes (TILs) secreting cytokines **(Figure 4J)**. Taken together, targeted delivery of anti-PD-L1 peptide and DOX by PD-NPs efficiently inhibit tumor progression by inducing strong antitumor immune responses, which are attributable to the effective ICD and PD-L1 blockade.

### Safety of ICB immunotherapy by PD-NPs

**Figure 5:**
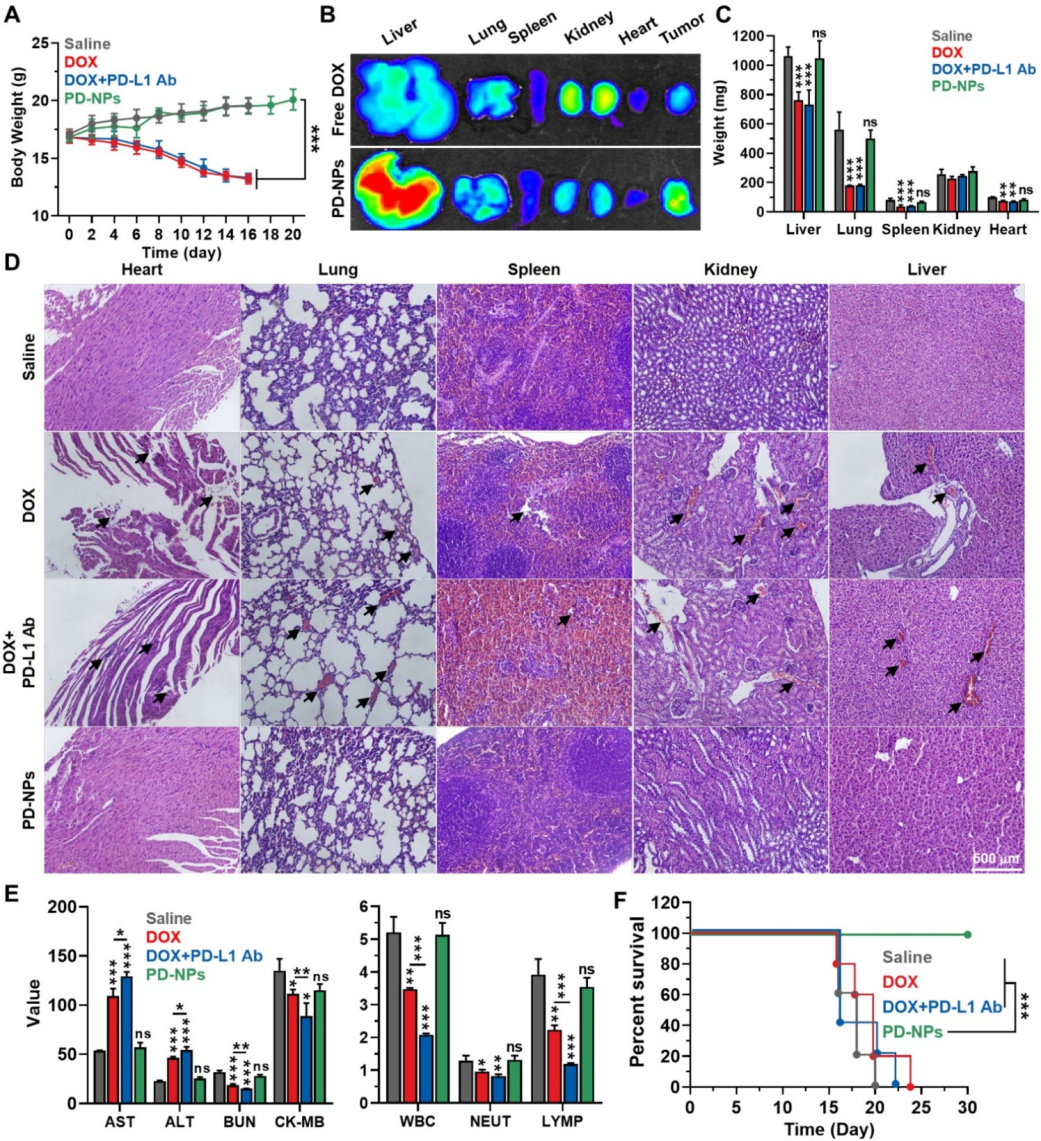
** Safety of ICB immunotherapy by PD-NPs. A.** Body weights during treatment with free DOX (3 mg/kg), free DOX with PD-L1 antibody (10 mg/kg), or PD-NPs (3 mg/kg based on DOX contents) once every three days.** B.** NIRF imaging of major organs from mice treated with free DOX or PD-NPs after 24 h injection.** C.** Organ weights after 16 days of treatment. **D.** Major organ tissues stained with H&E after 16 days of treatment. Black arrows indicate structural abnormalities. **E.** Blood analyses of mice treated with free DOX, free DOX with PD-L1 antibody, or PD-NPs once every three days. Blood samples were collected after 16 days of treatment. **F.** Survival analysis of mice. Significance was determined by one-way ANOVA with the Tukey-Kramer *posthoc* test (A, C, E) or log-rank test (F). The asterisk (*P < 0.05, **P < 0.01, ***P < 0.001) in Figures indicate comparison with Saline group (C, E).

The *in vivo* safety of PD-NPs based ICB immunotherapy was compared to free DOX or free DOX with anti-PD-L1 antibody in 4T1 tumor-bearing mice. The mice were treated once every three days as in **Figure 4**. First, the body weights of mice in the PD-NPs group were gradually increased during treatment and similar with saline group, whereas free DOX and free DOX with anti-PD-L1 antibody groups showed severe body weight loss owing to the *in vivo* systemic toxicity **(Figure 5A)**. After 24 h of injection, PD-NPs showed significantly higher accumulation in the liver tissues, and distribution in other organs was similar with free DOX as shown *in ex vivo* NIRF imaging **(Figure 5B and S16)**. However, their toxicity towards organs was greatly minimized by high cathepsin B-specificity, as confirmed by histological and hematological analyses, on the 16 day that occurred serious body weight loss in free DOX and free DOX with anti-PD-L1 antibody groups. The free DOX and free DOX with anti-PD-L1 antibody caused serious reduction of organ weights, while those in the PD-NPs group were similar with saline group **(Figure 5C)**. In addition, the major organs stained with H&E exhibited substantial damaged areas in free DOX and free DOX with anti-PD-L1 antibody groups, but significant structural abnormalities were not observed in the PD-NPs group **(Figure 5D)**. The blood analyses exhibited severe cardiac, renal and hepatic toxicity in the free DOX group, as confirmed by significant changes in the hematological parameters, such as aspartate aminotransferase (AST), alanine transaminase (ALT), blood urea nitrogen (BUN) and creatinine kinase (CK; **Figure 5E and S17)**. Importantly, organ toxicity more worsens in the free DOX with anti-PD-L1 antibody group, and severe leukopenia, neutropenia and lymphocytopenia were also observed. The extensive organ dysfunction accompanying hematological toxicity is representative immune-related adverse events (irAEs), thus these results show limitations of current ICB immunotherapy that is suffer from severe side effects 9, 31. In contrast, those hematological parameters in the PD-NPs group were in normal range and similar with saline group. In agreement with the above toxicity analyses, the median survival of free DOX and free DOX with anti-PD-L1 antibody groups was measured to be 20 and 16 days, respectively, whereas PD-NPs-treated mice were survived over 30 days **(Figure 5F)**. As a control, saline group showed the median survival of 18 days, wherein the mice were dead due to tumor progression. These findings demonstrate that PD-NPs efficiently mitigate the both of off-target toxicity and irAEs by chemotherapeutic drugs and mAbs for ICB immunotherapy.

## Conclusions

In summary, we proposed how overcoming the unfavorable limitations of current ICB immunotherapy with anti-PD-L1 peptide-conjugated prodrug nanoparticles (PD-NPs) *via* targeted tumor delivery of anti-PD-L1 peptide and DOX for combining PD-L1 blockade and ICD. The PD-NPs self-assembled of functional peptide-drug conjugates exhibited high and accurate drug loading contents and stable nanoparicle strcutre in physiological condition without additional carrier materials. Importantly, the PD-NPs selectively released free DOX in cathepsin B-overexpressed cancer cells after PD-L1-mediated endocytosis, which provoked strong antitumor immune responses through combining of PD-L1 blockade and ICD. The *in vivo* study further demonstrated that highly accumulated PD-NPs in the tumor tissues *via* EPR effect not only turn the immunosuppressive tumor microenvironment to the immune-responsive tumors by recruiting a large amount of immune cells through DOX-mediated ICD, but also enhance the pre-existing antitumor immune responses of T lymphocytes by anti-PD-L1 peptide-mediated blockade of PD-1/PD-L1 interactions. As a result, localized tumor delivery of anti-PD-L1 peptide and DOX by PD-NPs greatly inhibited the tumor progression with minimal side effects. This work provides an approach for effective and safe ICB immunotherapy, which may open avenues for advanced prodrug design and provide important insight into translational nanomedicine.

## Supplementary Material

Supplementary figures.

## Abbreviations

DOX: doxorubicin
ICB: immune checkpoint blockade
mAbs: monoclonal antibodies
PD-1: programmed death-receptor 1
PD-L1: programmed death-ligand 1
CTLA-4: cytotoxic T lymphocyte associated protein 4
DAMPs: damage-associated molecular patterns
DCs: dendritic cells
ITM: immunosuppressive tumor microenvironment
irAEs: immune-related adverse events
EPR: enhanced permeability and retention
CRT: calreticulin
HMGB1: high mobility group box 1
ATP: adenosine triphosphate
AST: aspartate transaminase
ALT: alanine aminotransferase
BUN: blood urea nitrogen
CK-MB: creatinine kinase-MB
WBC: white blood cell
NEUT: neutrophil
LYMP: lymphocytes
CBCs: complete blood counts
HPLC: highperformance liquid chromatography
DLS: dynamic light scattering
TEM: transmission electron microscopy
H&E: hematoxylin-eosin
TUNEL: terminal deoxynucleotidyl transferase dUTP nick end labeling
