# Supplementary material for: Anti-PD-L1 peptide-conjugated prodrug nanoparticles for targeted cancer immunotherapy combining PD-L1 blockade with immunogenic cell death
Source: Theranostics. 2022 Jan 31;12(5):1999–2014. doi: 10.7150/thno.69119 (PMC8899589; doi:10.7150/thno.69119)
Supplement: Supplementary file 1 — Supplementary figures. [file thnov12p1999s1.pdf]

Supplementary Information for

Anti-PD-L1 peptide-conjugated prodrug nanoparticles for targeted cancer immunotherapy combining PD-L1 blockade with immunogenic cell death

Yujeong Moon[1,2,#], Man Kyu Shim[1,#], Jiwoong Choi[1,3], Suah Yang[1,3], Jinseong Kim[1,3], Wan Su Yun[1,3], Hanhee Cho[1,4], Jung Yeon Park[3], Yongju Kim[3], Joon-Kyung Seong[2], Kwangmeyung Kim[1,3,*]

[1]Biomedical Research Institute, Korea Institute of Science and Technology (KIST), Seoul, 02792, Republic of Korea.

[2]Department of Bioengineering, Korea University, Seoul, 02841, Republic of Korea.

[3]KU-KIST Graduate School of Converging Science and Technology, Korea University, Seoul, 02841, Republic of Korea.

[4]Department of Materials Science and Engineering, Seoul National University, Seoul, 08826, Republic of Korea.

[#]These authors contributed equally to this work.

*Correspondence and requests for materials should be addressed to **K. Kim** (E-mail: kim@kist.re.kr; address: Biomedical Research Institute, Korea Institute of Science and Technology (KIST), Seoul, 02792, Republic of Korea).

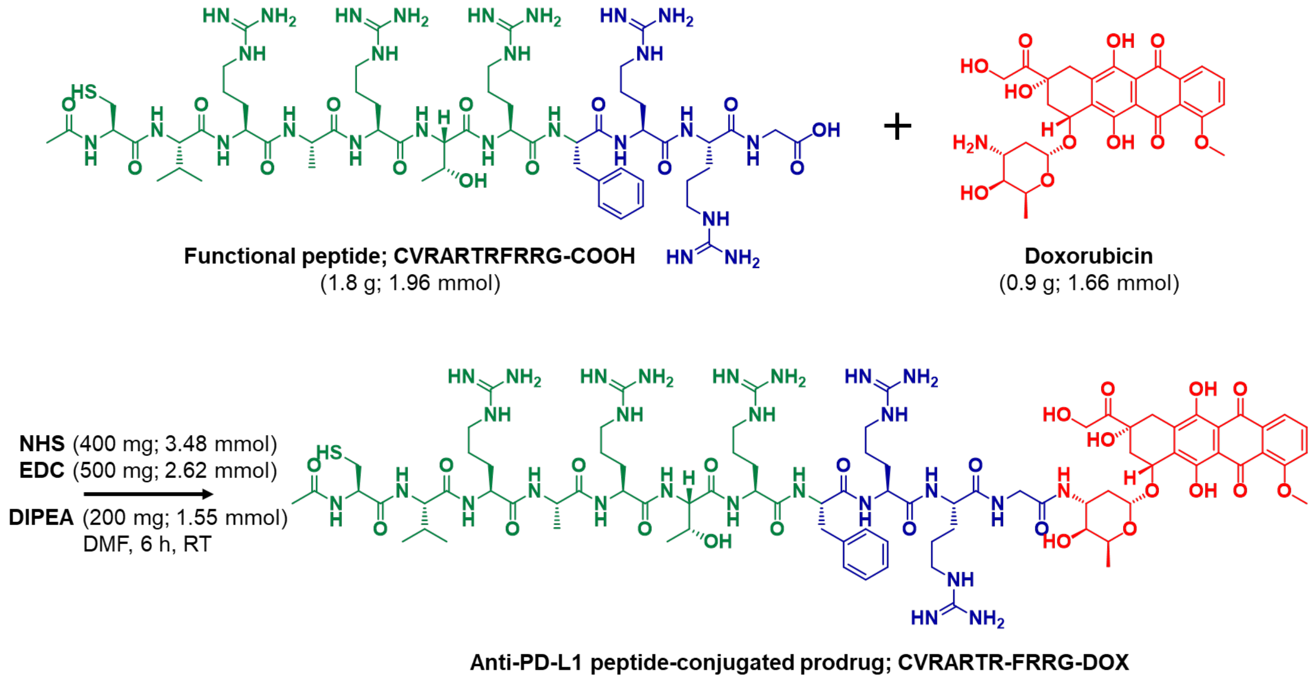

Figure S1. Synthetic scheme to prepare anti-PD-L1 peptide-conjugated prodrug (CVRARTRFRRG-DOX). First, Ac-CVRARTRFRRG-COOH (1.8 g, 1.96 mmol), DOX (0.9 g, 1.66 mmol), NHS (400 mg, 3.48 mmol) and EDC (500 mg, 2.62 mmol) were dissolved in anhydrous DMF (100 mL), and DIPEA (200 mg, 1.55 mmol) was subsequently added in mixture solution. After 6 h of incubation, prodrug was purified using high performance liquid chromatography (HPLC, Agilent Technologies, 1200 series, USA) and lyophilized at -90 ^{o}C and 5 mTorr for 24 h to obtain as a red powder (Freeze Dryer, ilShinBioBase, Republic of Korea).

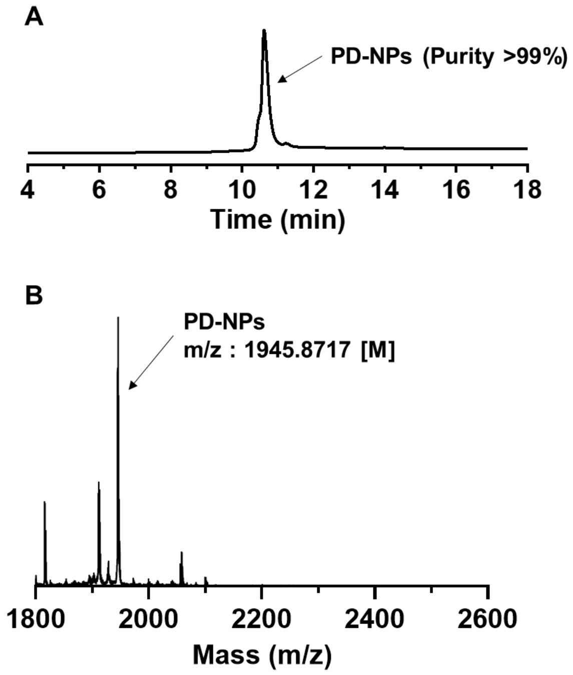

Figure S2. Characterization of anti-PD-L1 peptide-conjugated prodrug (CVRARTRFRRG-DOX). A. The purity (> 99%) of CVRARTRFRRG-DOX was confirmed by high performance liquid chromatography (HPLC). **B.** The molecular weight of CVRARTRFRRG-DOX was confirmed *via* MALDI-TOF mass spectrometer; molecular weight was calculated to be 1945.19 Da for $C_{85}H_{129}N_{27}O_{24}S$, and measured to be 1945.8717 m/z.

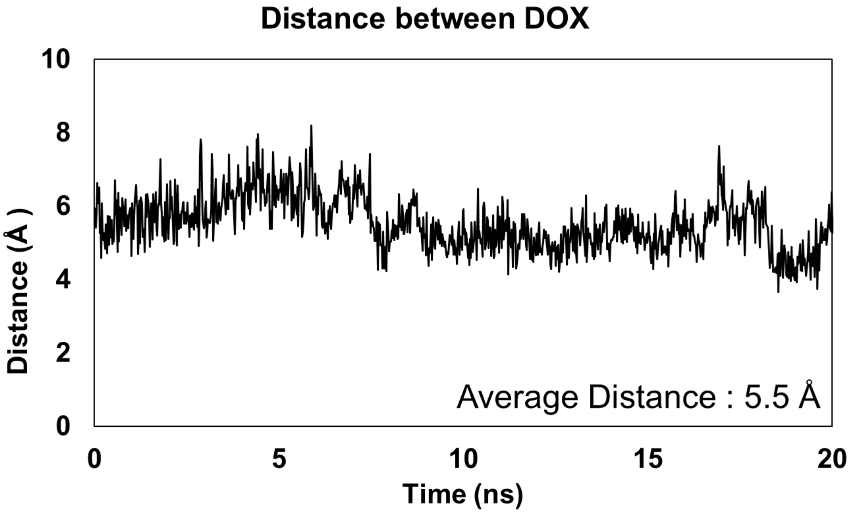

Figure S3. Molecular dynamics (MD) simulation result for average distance between DOX in two molecules of CVRARTRFRRG-DOX conjugates.

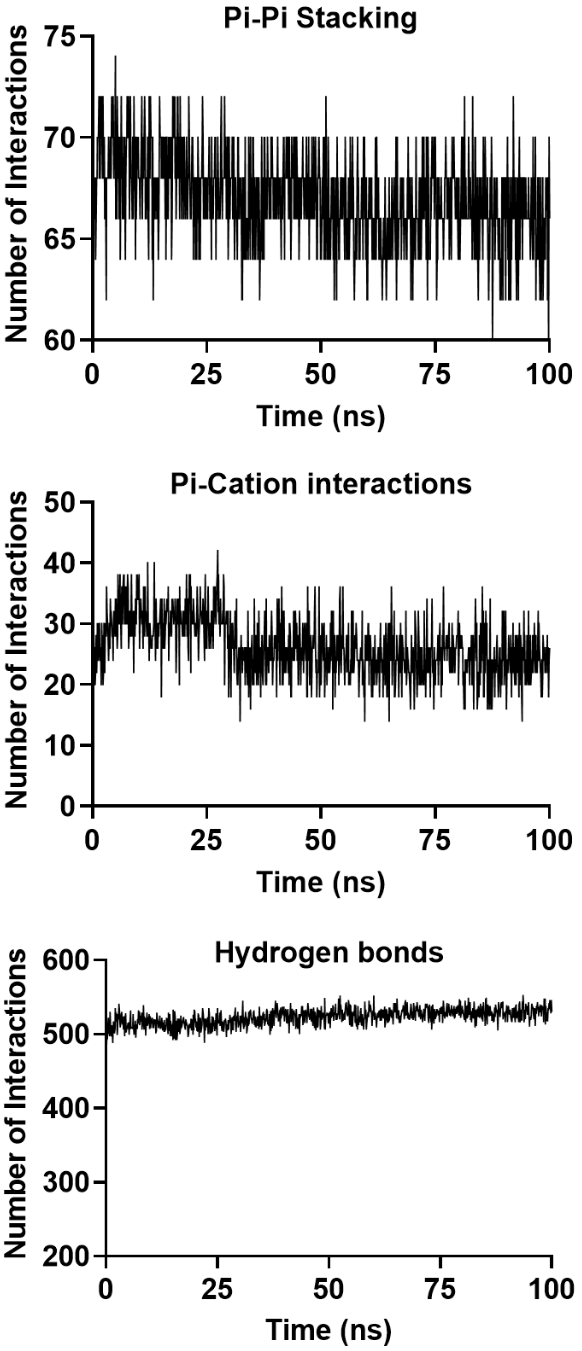

Figure S4. All atom MD simulation results showing driving force to promote self-assembly of twenty molecules of CVRARTRFRRG-DOX conjugates.

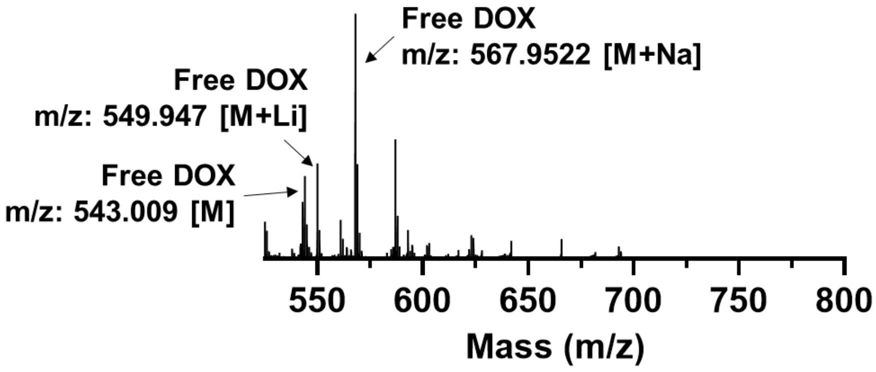

Figure S5. Metabolite assay of anti-PD-L1 peptide-conjugated prodrug nanoparticles (PD-NPs). Free DOX released from PD-NPs was confirmed *via* MALDI-TOF mass spectrometer. For this analysis, PD-NPs were incubated with cathepsin B (10 μg) in MES buffer (pH 5.5) for 24 h, and enzyme reaction buffer was analyzed. As a result, the molecular weights of free DOX were measured to be 543.009 m/z [M], 549.947 m/z [M+Li], 567.9522 m/z [M+Na].

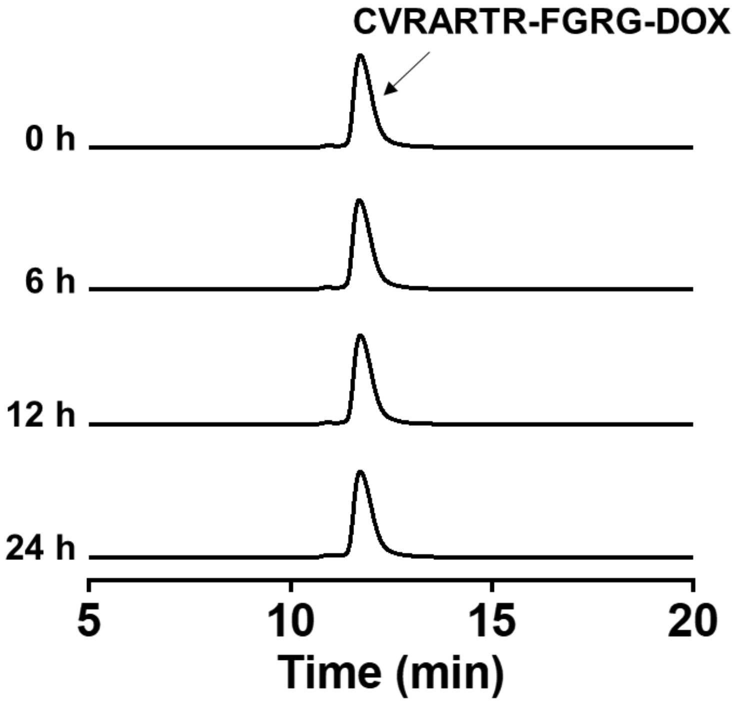

Figure S6. HPLC spectrum of CVRARTR-FGRG-DOX after 0, 6, 12 and 24 h of incubation in the presence of cathepsin B enzyme (10 μg) at acidic condition (pH 5.5 MES buffer); CVRARTR-FGRG-DOX that absence cathepsin B-specific cleavable peptide of RRG was not cleaved by cathepsin B.

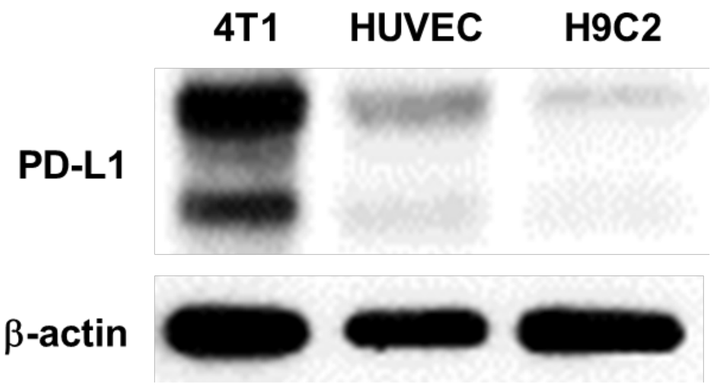

Figure S7. PD-L1 expression levels in 4T1, HUVEC and H9C2 cells.

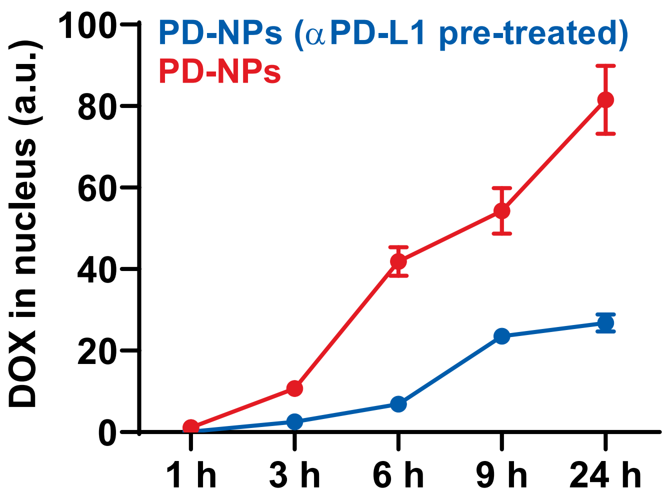

Figure S8. Quantification analysis on the amount of DOX fluorescence in nuclei of 4T1 or anti-PD-L1-treated 4T1 cells after PD-NPs treatment.

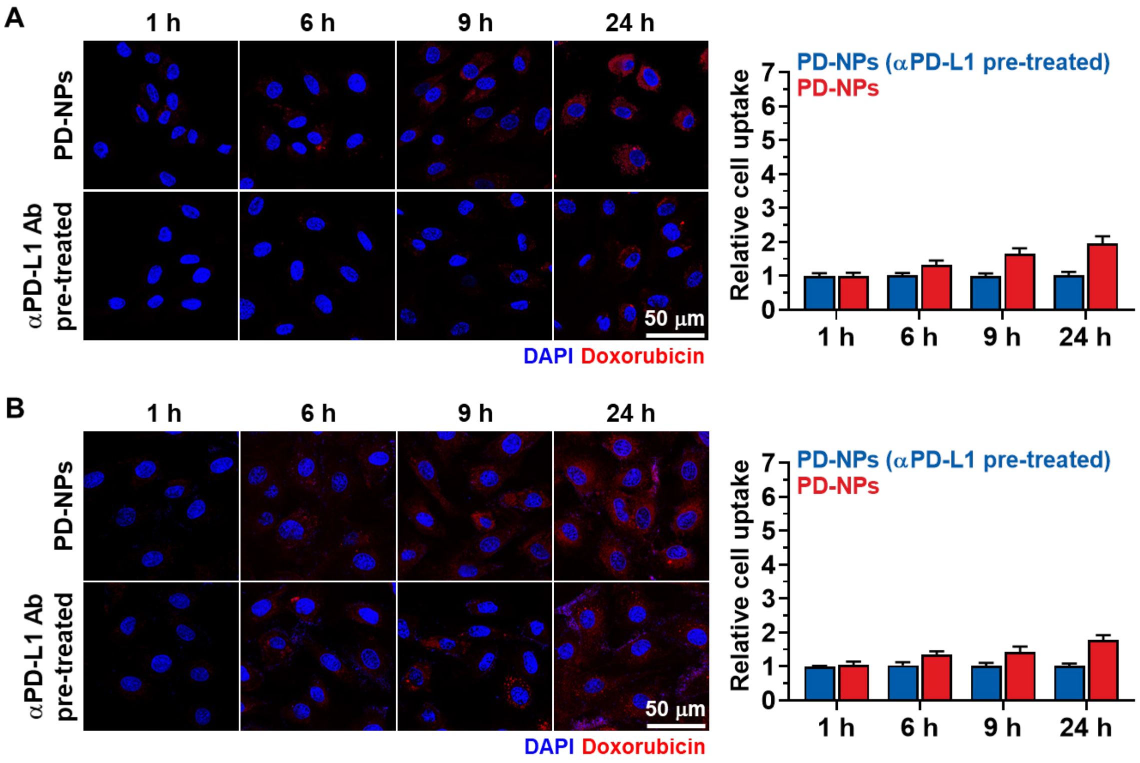

Figure S9. Time-dependent cellular uptake of PD-NPs in **A.** HUVEC and **B.** H9C2 cells. As a control, anti-PD-L1 antibody was pre-incubated with each cell for 24 h before PD-NPs treatment.

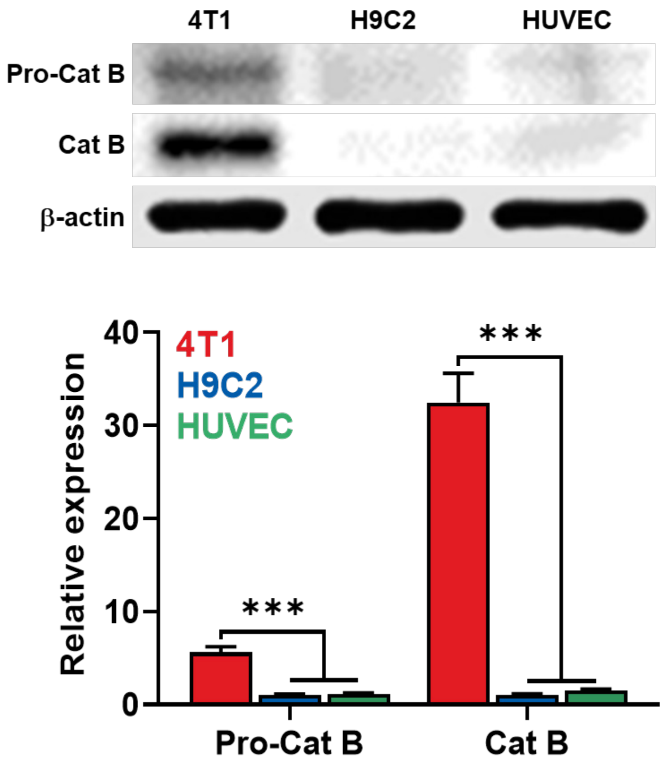

Figure S10. Cathepsin B expression levels in 4T1, HUVEC and H9C2 cells. Relative expression levels of pro-cathepsin B (Pro-Cat B) and cathepsin B (Cat B) in cells; 4T1 cells expressed 32.48 ± 3.14-fold and 26.13 ± 2.78-fold of Cat B, and 5.62 ± 0.581-fold and 4.39 ± 0.41-fold of Pro-Cat B, compared to H9C2 and HUVEC cells, respectively.

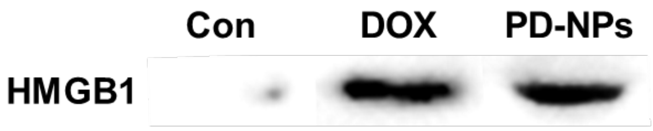

Figure S11. Western blot analysis of released HMGB1 from 4T1 cells. After 24 h treatment of 4T1 cells with free DOX or PD-NPs (2 μM), HMGB1 in cell culture medium was analyzed *via* western blot analysis.

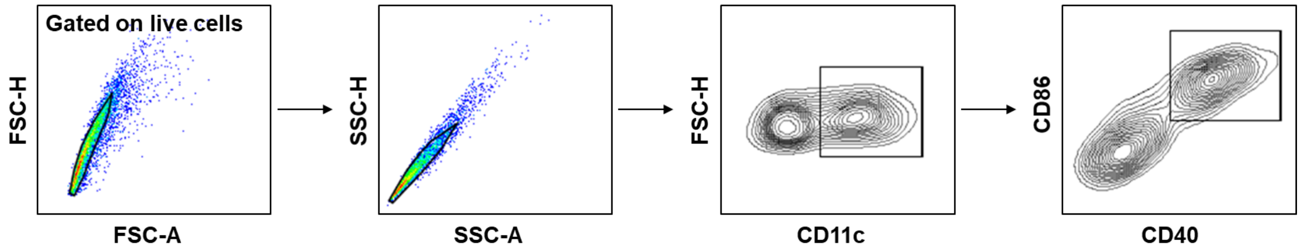

Figure S12. The flow cytometric gating strategy to analyze the mature DCs after BMDCs were co-cultured with DAMPs released from 4T1 cells treated with free DOX or PD-NPs (2 μM). FSC x SSC gating was used to obtain live cells and singlets based on granularity and size. The mature DCs were analyzed after staining with CD11c, CD40 and CD86.

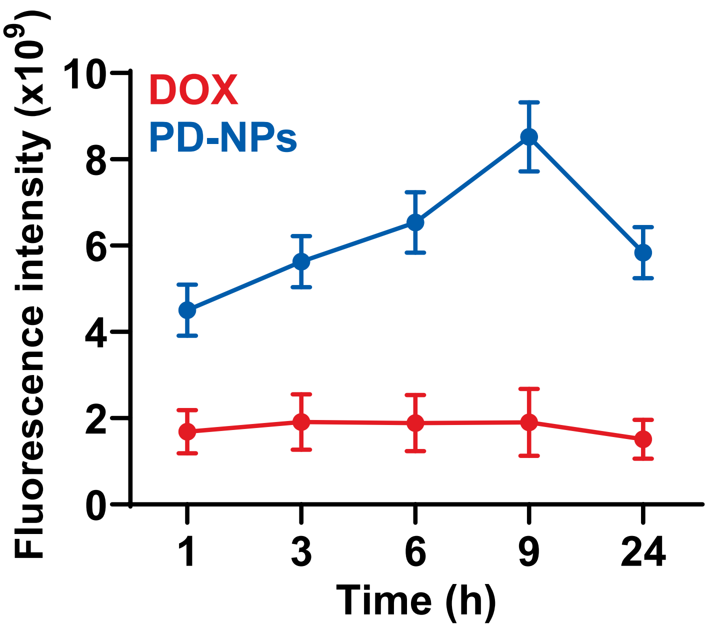

Figure S13. Quantification analysis on the DOX fluorescence at tumor tissues of mice treated with free DOX or PD-NPs (3 mg/kg based on DOX contents).

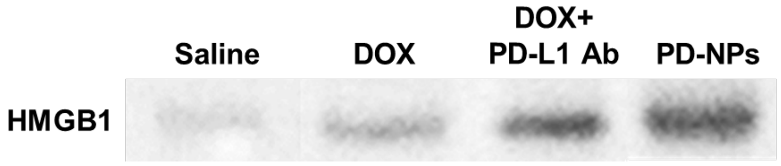

Figure S14. Released HMGB1 into tumor microenvironment was analyzed *via* western blot analysis. For this analysis, 4T1 tumor-bearing mice were treated with free DOX (3 mg/kg), free DOX with anti-PD-L1 antibody (10 mg/kg), or PD-NPs (3 mg/kg based on DOX contents) once every three days. On 7 days after treatment, HMGB1 in tumor supernatants was analyzed *via* western blot analysis.

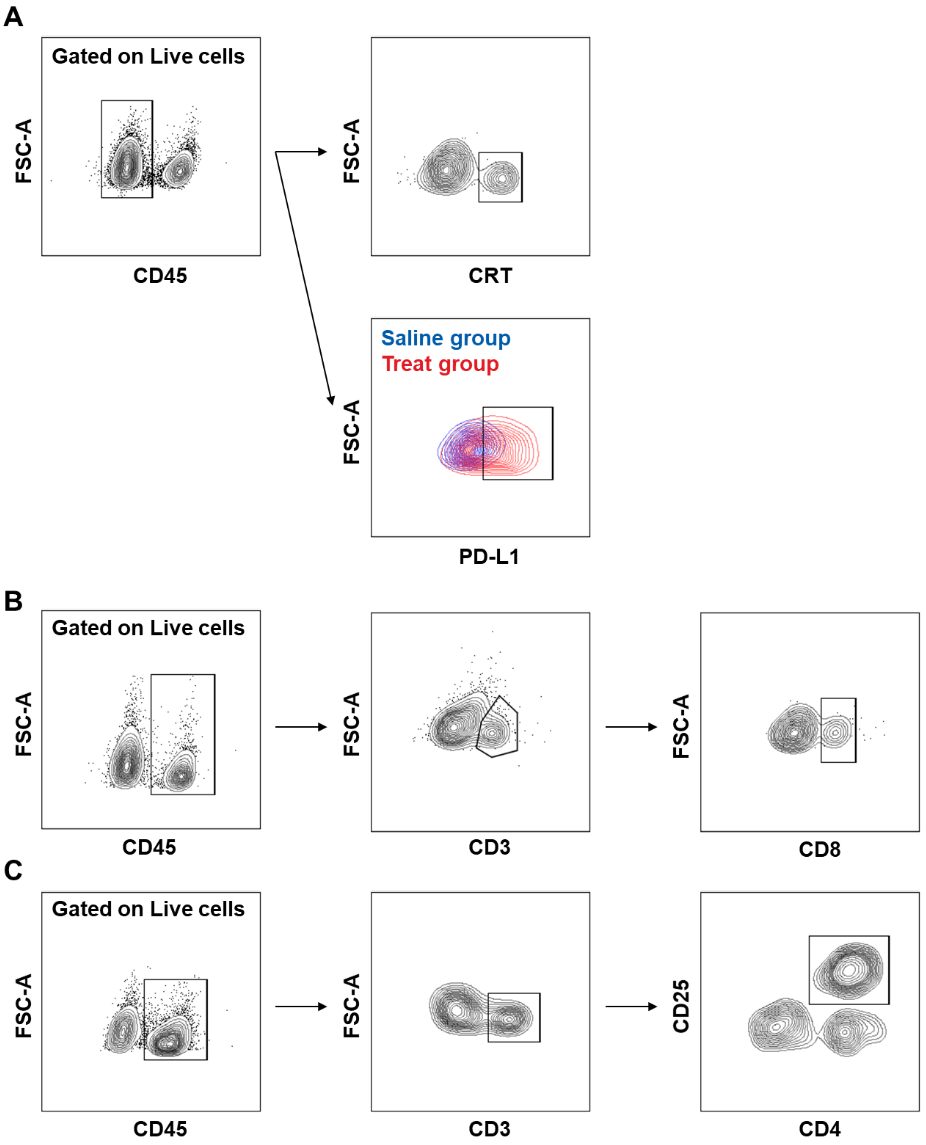

Figure S15. The flow cytometric gating strategy to analyze the immune cells in tumor tissues after treatment of mice with free DOX (3 mg/kg), free DOX with anti-PD-L1 antibody (10 mg/kg) or PD-NPs (3 mg/kg based on DOX contents) once every three days. On 7 days after treatment, tumor tissues were collected from mice and analyzed *via* flow cytometry. FSC x SSC gating was used to obtain live cells and singlets based on granularity and size. CD45 was used as a leukocyte marker. **A.** CRT positive cancer cells ($CD45^{-}CRT^{+}$) and PD-L1 positive cancer cells ($CD45^{-}PD\text{-}L1^{+}$), **B.** cytotoxic T lymphocytes ($CD45^{+}CD3^{+}CD8^{+}$) and **C.** regulatory T lymphocytes ($CD45^{+}CD3^{+}CD4^{+}CD25^{+}$) were analyzed.

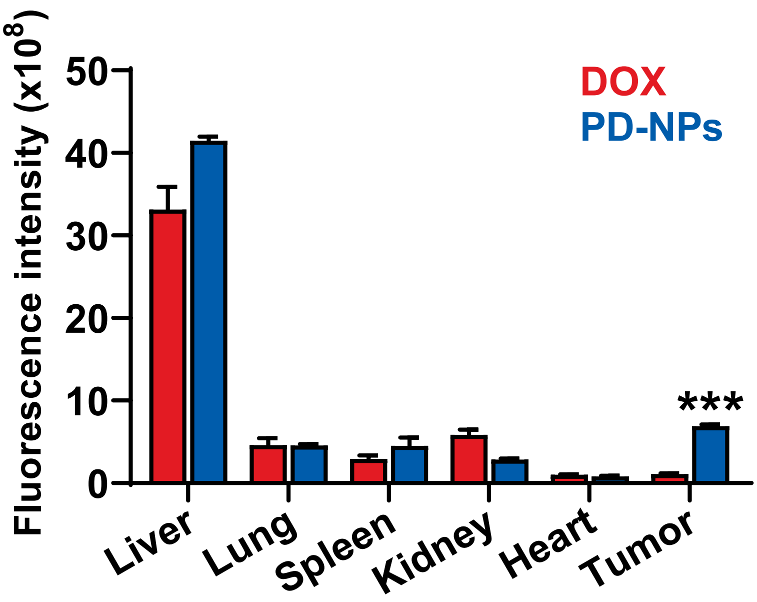

Figure S16. Quantification analysis on the DOX fluorescence in major organs of mice treated with free DOX or PD-NPs (3 mg/kg based on DOX contents) after 24 h of injection.

Parameters	Saline	DOX	DOX+PD-L1 Ab	PD-NPs
**AST (U/L)**	53.67 ± 0.42	109.07 ± 7.71	129.37 ± 4.43	57.23 ± 4.68
**ALT (U/L)**	22.83 ± 0.58	46.2 ± 1.56	54.23 ± 3.42	25.47 ± 1.53
**BUN (mg/dl)**	31.89 ± 1.7	18.74 ± 1.12	15.04 ± 0.27	27.96 ± 1.28
**CK-MB (U/L)**	135.03 ± 12.12	111.37 ± 4.31	88.67 ± 13.35	115.33 ± 6.12
**WBC (10^3/μL)**	5.21 ± 0.48	3.48 ± 0.03	2.08 ± 0.04	5.135 ± 0.36
**NEUT (10^3/μL)**	1.29 ± 0.16	0.96 ± 0.06	0.82 ± 0.06	1.315 ± 0.13
**LYMPH (10^3/μL)**	3.92 ± 0.48	2.23 ± 1.14	1.19 ± 0.04	3.55 ± 0.28

Figure S17. Detail information of blood analysis results. For these analyses, 4T1 tumor-bearing mice were treated with free DOX (3 mg/kg), free DOX with anti-PD-L1 antibody (10 mg/kg) or PD-NPs (3 mg/kg based on DOX contents) once every three days. On 16 days after treatment, following hematological parameters in blood samples from mice were analyzed. AST, aspartate aminotransferase; ALT, alanine transaminase; BUN, blood urea nitrogen; CK-MB, creatine kinase MB isoenzyme; WBC, white blood cell; NEUT, neutrophil; and LYMP, lymphocytes.
